# Deciphering the scalene association among type‐2 diabetes mellitus, prostate cancer, and chronic myeloid leukemia via enrichment analysis of disease‐gene network

**DOI:** 10.1002/cam4.1845

**Published:** 2019-04-01

**Authors:** Qiong Liu, Yingying Zhang, Pengqian Wang, Jun Liu, Bing Li, Yanan Yu, Hongli Wu, Ruixia Kang, Xiaoxu Zhang, Zhong Wang

**Affiliations:** ^1^ Institute of Basic Research in Clinical Medicine China Academy of Chinese Medical Sciences Beijing China; ^2^ Dongzhimen Hospital Beijing University of Chinese Medicine Beijing China; ^3^ Institute of Chinese Materia Medica China Academy of Chinese Medical Sciences Beijing China; ^4^ Institute of Information on Traditional Chinese Medicine China Academy of Chinese Medical Sciences Beijing China; ^5^ Eye Hospital China Academy of Chinese Medical Sciences Beijing China

**Keywords:** chronic myeloid leukemia, overlapping gene and module, prostate cancer, therapeutic prediction, type 2 diabetes mellitus

## Abstract

The potential biological relationship between type‐2 diabetes mellitus (T2DM) has been focused in numerous studies. To investigate the molecular associations among T2DM, prostate cancer (PCa), and chronic myeloid leukemia (CML), using a biomolecular network enrichment analysis. We obtained a list of disease‐related genes and constructed disease networks. Then, GO enrichment analysis was performed to identify the significant functions and pathways of overlapping modules in the Database for Annotation, Visualization and Integrated Discovery (DAVID) database. More than 75% of these overlapping genes were found to be consistent with the findings of previous studies. In the three diseases, we found that Sarcoglycan delta (SGCD) and Rho family GTPase 3 (RND3) were the overlapping genes and identified negative regulation of apoptotic process and negative regulation of transcription from RNA polymerase II promoter RNA as the two overlapping biological functions. CML and PCa were the most closely related, with 34 overlapping genes, five overlapping modules, 27 overlapping biological functions, and nine overlapping pathways. There were 13 overlapping genes, one overlapping modules, four overlapping biological functions and one overlapping pathway (FoxO signaling pathway) were found in T2DM and CML.And T2DM and PCa were the least related pair in our study, with only six overlapping genes, five overlapping modules, and one overlapping biological function. SGCD and RND3 were the main gene‐to‐gene relationship among T2DM, CML, and PCa; apoptosis, development, and transcription from RNA polymerase II promote processes were the main functional connections among T2DM, CML, and PCa by network enrichment analysis. There is a “scalene” relationship among T2DM, CML, and PCa at gene, pathway, biological process, and module levels: CML and PCa were the most closely related, the second were T2DM and PCa, and T2DM and PCa were the least related pair in our study. Our study provides a new avenue for further studies on T2DM and cancers, which may promote the discovery and development of novel therapeutic and can be used to treat multiple diseases.

## 1 INTRODUCTION

Type 2 diabetes mellitus (T2DM), a substantial worldwide health problem, is a heterogeneous disorder caused by complex interplay between genetic and environmental factors. According to the International Diabetes Federation (IDF), more than 640 million people worldwide will suffer from diabetes by 2040.^1^ Prostate cancer (PCa) has become one of the most common types of cancer in men, and its increasing incidence and mortality seriously affect the health of the elderly male population.^2^ Chronic myeloid leukemia (CML) is a type of white blood cell cancer characterized by increased proliferation of granulocytes, which was the first cancer to be linked to a clear genetic abnormality.^3^, ^4^ To date, the potential relationship between cancer and T2DM has been examined in numerous epidemiological studies and has become a critically important area of study in both developing and developed countries. It has been shown in some studies that T2DM patients are more likely to suffer from PCa and may benefit from increased surveillance for PCa.^5^ However, there is no direct evidence to show the correlations among these three diseases. Therefore, we would like to investigate the potential molecular correlations among the three diseases by network enrichment analysis.

Finding gene‐disease and disease‐disease associations play important roles in the biomedical area, and many prioritization methods have been proposed for this goal.^6^ Enrichment analysis has gained acceptance as a method to characterize both modest and robust, coordinated, biologically relevant changes in molecular signaling pathways.^7^ The advances in systems biology have led to an increased interest in systems‐oriented network enrichment analysis appears to be a promising tool for systems biology studies.^8^, ^9^ By analyzing modules and their functional annotations, a prior study identified atherosclerosis, cholesterol homeostasis, plasma lipoprotein particle remodeling and response to oxidative stress as the potential risk factors for coronary heart disease (CHD) and stroke.^10^ Based on network pharmacological analysis, a study by Zhang et al^11^ discovered the new biological functions of dopamine receptors and verified the molecular mechanisms of cell death related to both diabetes and breast cancer. Another study by Yuan et al^12^ showed that through network construction and modular analysis based on disease‐related genes, glycosphingolipid biosynthesis, arachidonic acid metabolism, and biosynthetic process might be the main pathways or functions involved in CHD, idiopathic pulmonary arterial hypertension (IPAH), and pulmonary heart disease (PHD).

In our study, firstly, we obtained a list of disease‐related genes form the Disease‐Connect website and we constructed disease networks by Agilent Literature Search software. Then, the disease networks were divided into modules by MCODE. GO enrichment analysis was performed to identify the significant functions and pathways of overlapping modules in the Database for Annotation, Visualization and Integrated Discovery (DAVID) database. Based on molecular network construction and enrichment analysis, our study aimed to systemically explore the molecular associations among T2DM, CML, and PCa, which provided a novel and feasible idea for the study the association in T2DM and different cancers.

## 2 MATERIALS AND METHODS

### 2.1 Obtaining the genes and GO enrichment analysis

The terms “type 2 diabetes mellitus (T2DM),” “chronic myeloid leukemia (CML),” and “malignant prostate cancer (PCa)” were entered into the “Disease View” search box of the Disease‐Connect website (https://disease-connect.org/), a freely available web database for mechanism‐based disease‐disease connections. We analyzed the functions of the disease‐related genes by using the Database for Annotation, Visualization and Integrated Discovery (DAVID) (https://david.abcc.ncifcrf.gov/), which provides a comprehensive set of functional annotation tools for understanding the biological significances of many genes.^13^ The following parameters were used: Count = 2; EASE = 0.01; and species and background = Homo sapiens.^11^ We conducted the Kyoto Encyclopedia of Genes and Genomes (KEGG) pathway and gene ontology (GO) enrichment analysis to identify the biological processes and pathways corresponding to the genes.^14^ A *P* < 0.05 was used as the cutoff criterion for GO and KEGG pathway enrichment analyses, and *P*‐values were ranked.

### 2.2 Constructing the networks for T2DM, CML, and PCa

Then, the disease‐related genes were submitted to Agilent Literature Search software, version 3.20 (https://www.cytoscape.org/), and an overview network of gene/protein associations was obtained. Cytoscape is an open source tool for visualizing complex networks and integrating biomolecular interaction networks, especially for large data sets of gene‐gene, gene‐protein, and protein‐protein interactions.^15^

### 2.3 Dividing the modules of networks

We used the Cytoscape software version 3.20 (https://www.cytoscape.org) to visualize the three disease‐related networks and analyze the properties of these networks. Network parameters (such as Clustering Coefficient, Network Diameter, Network Centralization, and Network Radius) were calculated and illustrated.^12^

### 2.4 Identification of modules

After constructing the networks for the three diseases, centrality analysis and a complex molecular algorithm MCODE 1.32 (https://baderlab.org/Software/MCODE) were performed for network module division.^16^ The parameters (Degree Cutoff = 2, Connectivity Threshold = 2, Node Score Cutoff = 0.2, Core Threshold *K* = 2, Node Score Threshold = 0.2, Max. Depth = 100) were used as the criteria for network module screening.^17^

### 2.5 Function and pathway enrichment analyses of overlapping modules

We analyzed the functions of the overlapping modules included in T2DM‐, CML‐, and PCa‐related gene networks. The following parameters were used: Count = 2; EASE = 0.01; and species and background = Homo sapiens.^10^ We conducted the KEGG pathway and GO enrichment analysis to identify the biological processes and pathways corresponding to these modules.^14^ A *P* < 0.05 was used as the cutoff criterion for GO and KEGG pathway enrichment analyses, and *P*‐values were ranked.

### 2.6 Validation of overlapping genes by literature‐based text mining

The overlapping genes in T2DM, CML, and PCa were provided by the Disease‐Connect website and the Agilent Literature Search in the Cytoscape system based on text mining. To validate the overlapping genes from literature, TKIs, T2DM, CML, PCa, and the gene IDs were the key words of searching in the PubMed database.

## 3 RESULTS

### 3.1 Disease‐related genes of T2DM, CML, and PCa in the disease‐connect database

**Figure 1:**
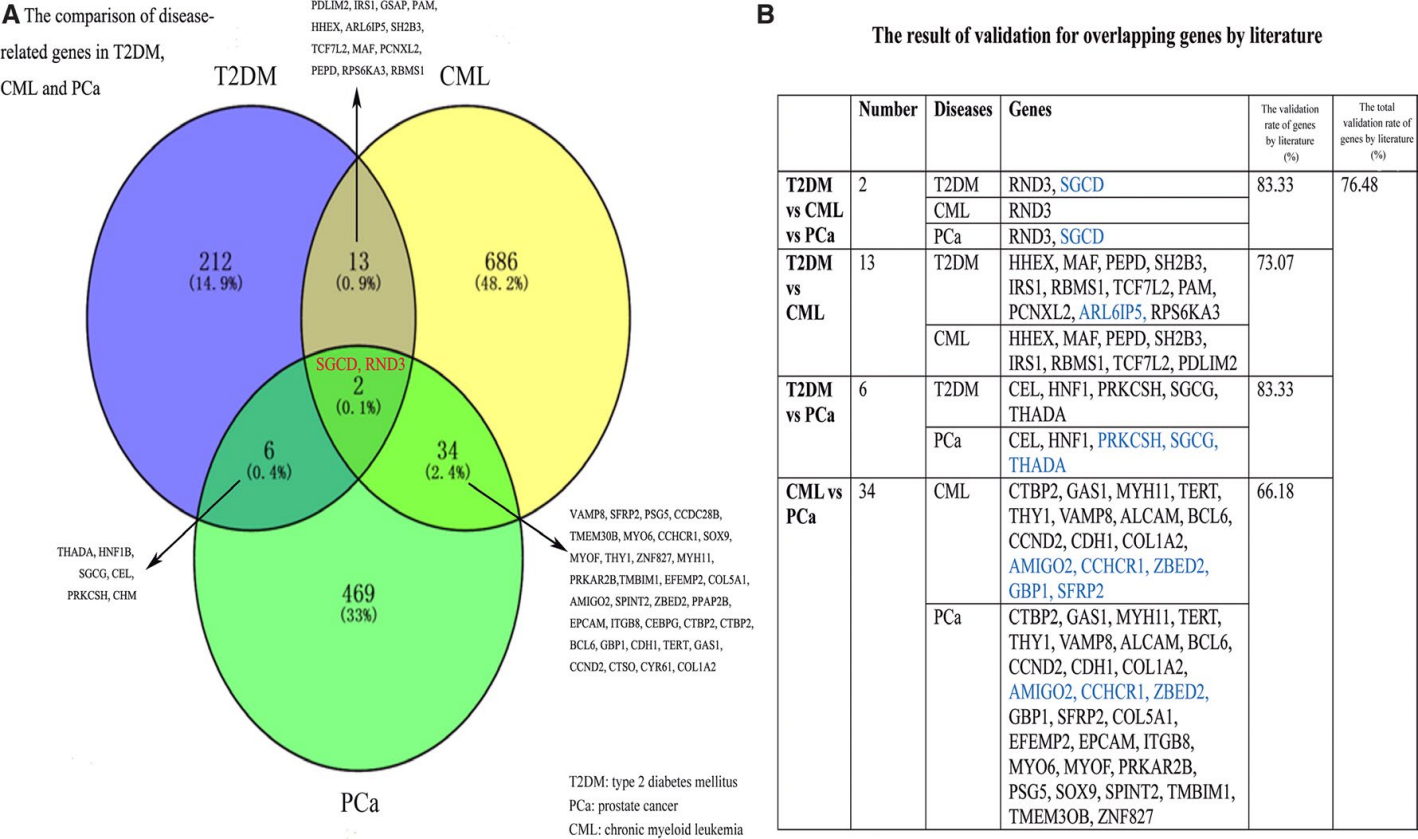
The comparison of disease‐related genes in T2DM, CML, and PCa. A, The comparison of disease‐related genes in T2DM, CML, and PCa. B, The results of validation for overlapping genes by literature (blue represents the new findings in our study)

Subsequent to searching the Disease‐Connect database (on May 10, 2016), we obtained 233 T2DM‐related, 733 CML‐related, and 511 PCa‐related genes (Table S1). SGCD and RND3 were found to be the overlapping genes among T2DM, CML and PCa. And 13 overlapping genes were detected between T2DM‐ and CML‐related genes, six between T2DM‐ and PCa‐related genes, 34 between CML‐ and PCa‐related genes, respectively (Figure 1A).

### 3.2 GO functional enrichment analysis of disease‐related genes

**Figure 2:**
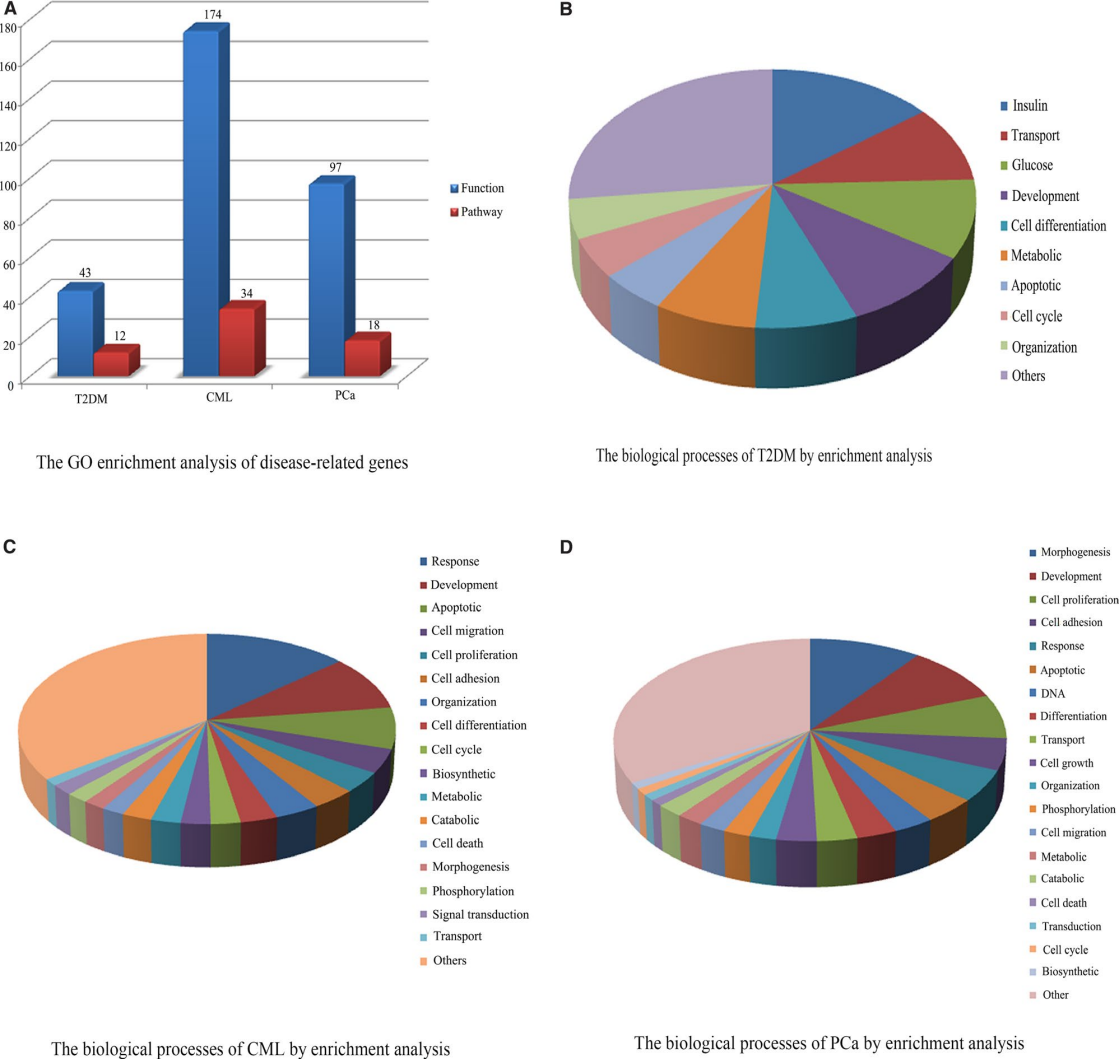
The GO enrichment analysis of disease‐related genes in T2DM, CML, and PCa. A, The GO enrichment analysis of disease‐related genes. B, The biological processes of T2DM by enrichment analysis. C, The biological processes of CML by enrichment analysis. D, The biological processes of PCa by enrichment analysis

All of the 233 T2DM‐related genes, 733 CML‐related genes, and 511 PCa‐related genes were submitted to DAVID for GO functional enrichment analysis. And we obtained the biological functions and pathways of these disease‐related genes. A total of 43 biological functions and 12 KEGG pathways were identified for the T2DM‐related genes, 174 biological functions and 34 KEGG pathways for the CML‐related genes, and 97 biological functions and 18 KEGG pathways for the PCa‐related genes, respectively (Figure 2A; Tables S2, S3 and S4).

In T2DM, insulin process (13.95%), transport process (9.3%), glucose process (9.3%), development process (9.3%), cell differentiation process (6.98%), and metabolic process (6.98%) were the main functional biological processes (Figure 2B). Response process (13.22%), development process (9.20%), apoptotic process (6.90%), cell migration (4.02%), cell proliferation process (3.45%), cell adhesion (3.45%), and organization (3.45%) accounted for most of the functional annotations in CML (Figure 2C). In PCa, morphogenesis process (10.31%), development process (9.28%), cell proliferation process (7.22%), cell adhesion process (5.15%), and response process (5.15%) were noted to be the major functional annotations (Figure 2D).

Two overlapping biological functions (negative regulation of apoptotic process, and negative regulation of transcription from RNA polymerase II promoter) were found among T2DM, CML, and PCa (Table S5). Four overlapping biological functions (response to drug, cytoskeleton organization, response to glucose, and fat cell differentiation) were discovered between CML and T2DM; endocrine pancreas development was the only overlapping function between T2DM and PCa; and 27 overlapping biological functions were observed between CML and PCa (Table S5).

No overlapping pathways were identified for the three diseases. FoxO signaling pathway was the overlapping pathway between T2DM and CML; nine overlapping pathways were found between CML and PCa, mainly related to cancer and cell adhesion (Table S5). And there were no overlapping pathways between T2DM and PCa.

### 3.3 Network construction of disease‐related genes in T2DM, CML, and PCa

**Figure 3:**
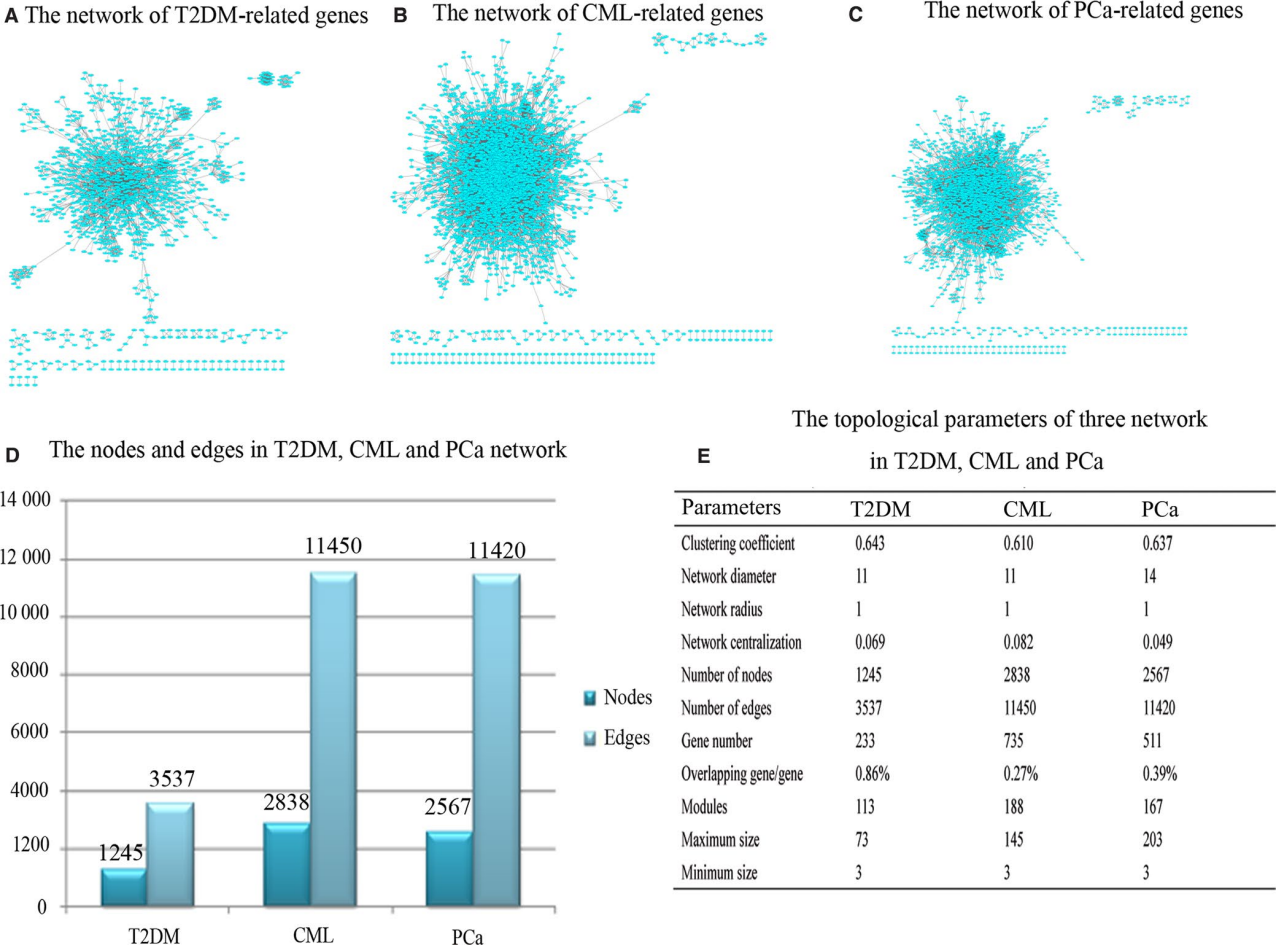
The network analysis of T2DM, CML, and PCa. A, The network of T2DM‐related genes. B, The network of CML‐related genes. C, The network of PCa‐related genes. D, The nodes and edges in T2DM, CML, and PCa networks. E, The topological parameters of the three networks

The disease‐related genes were submitted to the Agilent Literature Search 3.2, and we obtained the T2DM‐related (Figure 3A), CML‐related (Figure 3B), and PCa‐related gene networks (Figure 3C). Totally, 1245 nodes (genes) and 3537 edges (interactions) were identified for the T2DM‐related genes, 2838 nodes (genes) and 11 450 edges (interactions) for the CML‐related genes, and 2567 nodes (genes) and 11 420 edges (interactions) for the PCa‐related genes, respectively (Figure 3D). Four hundred and fifty‐six overlapping nodes were discovered among the three networks, and 240, 141, and 942 overlapping nodes were obtained between T2DM‐ and CML‐related networks, T2DM‐ and PCa‐related networks, and CML‐ and PCa‐related networks, respectively. We noted that these overlapping nodes accounted for 36.63% (456/1245) of the identified T2DM‐related nodes, 16.07% (456/2838) of CML‐related nodes, and 17.76% (456/2567) of PCa‐related nodes (Figure 3E). The network topological attributes and modularity of the three diseases were shown in Figure 3E.

### 3.4 Modularity analysis

**Figure 4:**
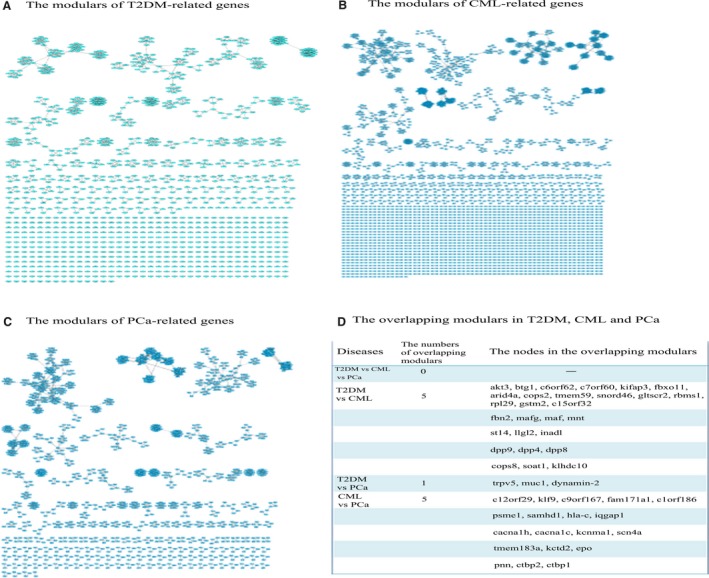
The modules of disease‐related genes in T2DM, CML, and PCa. A, The modules of T2DM‐related genes. B, The modules of CML‐related genes. C, The modules of PCa‐related genes

By using the plug‐in MCODE v1.32, 112 modules were identified from the T2DM‐related gene network (Figure 4A), 186 modules from the CML‐related gene network (Figure 4B), and 164 modules from the PCa‐related gene network (Figure 4C). The top 10 modules were ranked by the MCODE score (Density*#Nodes) for the T2DM‐, CML‐, and PCa‐related gene networks (Tables S6, S7 and S8). No overlapping functional modules were identified for the three networks, but 5, 1, and 5 overlapping modules were found between T2DM and CML, T2DM and PCa, and CML and PCa, respectively (Figure 4D).

### 3.5 GO functional enrichment analysis of modules

#### 3.5.1 GO functional enrichment analysis of RND3 and SGCD modules

**Figure 5:**
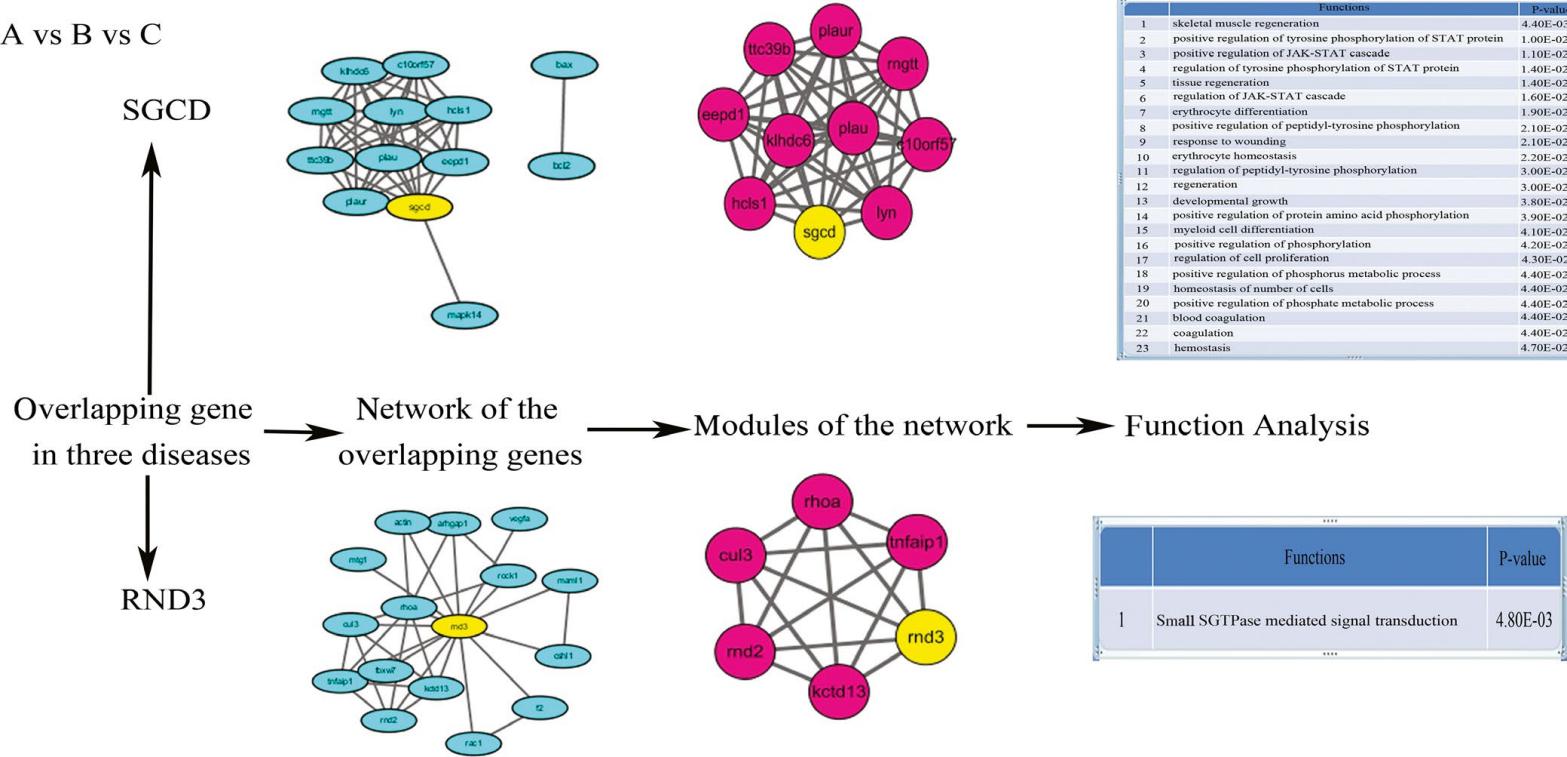
The network and enrichment analysis of RND3 and SGCD

Based on the overlapping genes (RND3 and SGCD) of the three diseases, we identified the modules for RND3 and SGCD by MCODE software, respectively. Functional analysis of the RND3 and SGCD modules by GO annotation was shown in Figure 5. Signal transducer and activator of transcription (STAT) protein process (17.39%), phosphorylation process (17.39%), and regeneration process (13.04%) were the main biological processes of SGCD, and small GTPase‐mediated signal transduction was the only functional process of RND3 (Figure 5).

#### 3.5.2 GO functional enrichment analysis of five overlapping modules between T2DM and CML

Proteolysis, transcription from RNA polymerase II promoter, cullin deneddylation, negative regulation of cell proliferation, nucleotide‐excision repair, DNA damage recognition, and neurogenesis were obtained by the GO functional enrichment analysis for the five overlapping modules between T2DM and CML (Table S9).

#### 3.5.3 GO functional enrichment analysis of five overlapping modules between CML and PCa

Regulation of ion transmembrane transport, white fat cell differentiation, viral genome replication, and membrane depolarization during action potential were the statistically significant functions of the five overlapping modules between CML and PCa (Table S9).

## 4 DISCUSSION

In our study, several new common biological backgrounds and overlapping genes of T2DM, CML, and PCa were discovered by network and enrichment analysis. In order to reveal and verify the genetic connections among T2DM, CML, and PCa, literature‐based text mining was performed. We discovered that T2DM and cancers have a certain molecular association at gene, pathway, biological process, and module levels according to network and enrichment analysis. More than 75% of the overlapping genes were found to be consistent with the findings of previous studies (Figure 1B). Hematopoietically expressed homeobox (HHEX), an overlapping gene between T2DM and CML, was significantly associated with T2DM and was found to inhibit Vascular endothelial growth factor (VEGF) signaling and promote cell survival in CML according to published literatures.^18^

RND3 and SGCD were the overlapping genes of the three diseases, which has also been reported in previous studies.^19^, ^20^, ^21^, ^22^, ^23^ Negative regulation of apoptotic process and negative regulation of transcription from RNA polymerase II promoter RNA were identified as the two overlapping biological functions among T2DM, CML, and PCa, which may provide novel insights into future studies. The previous studies suggested that patients with T2DM via apoptotic process in inflammation regulation.^24^ Moustafa et al^25^ found that dysregulated miRNA signature mapped to key regulatory factors involved in tumorigenesis, including apoptosis.

**Figure 6:**
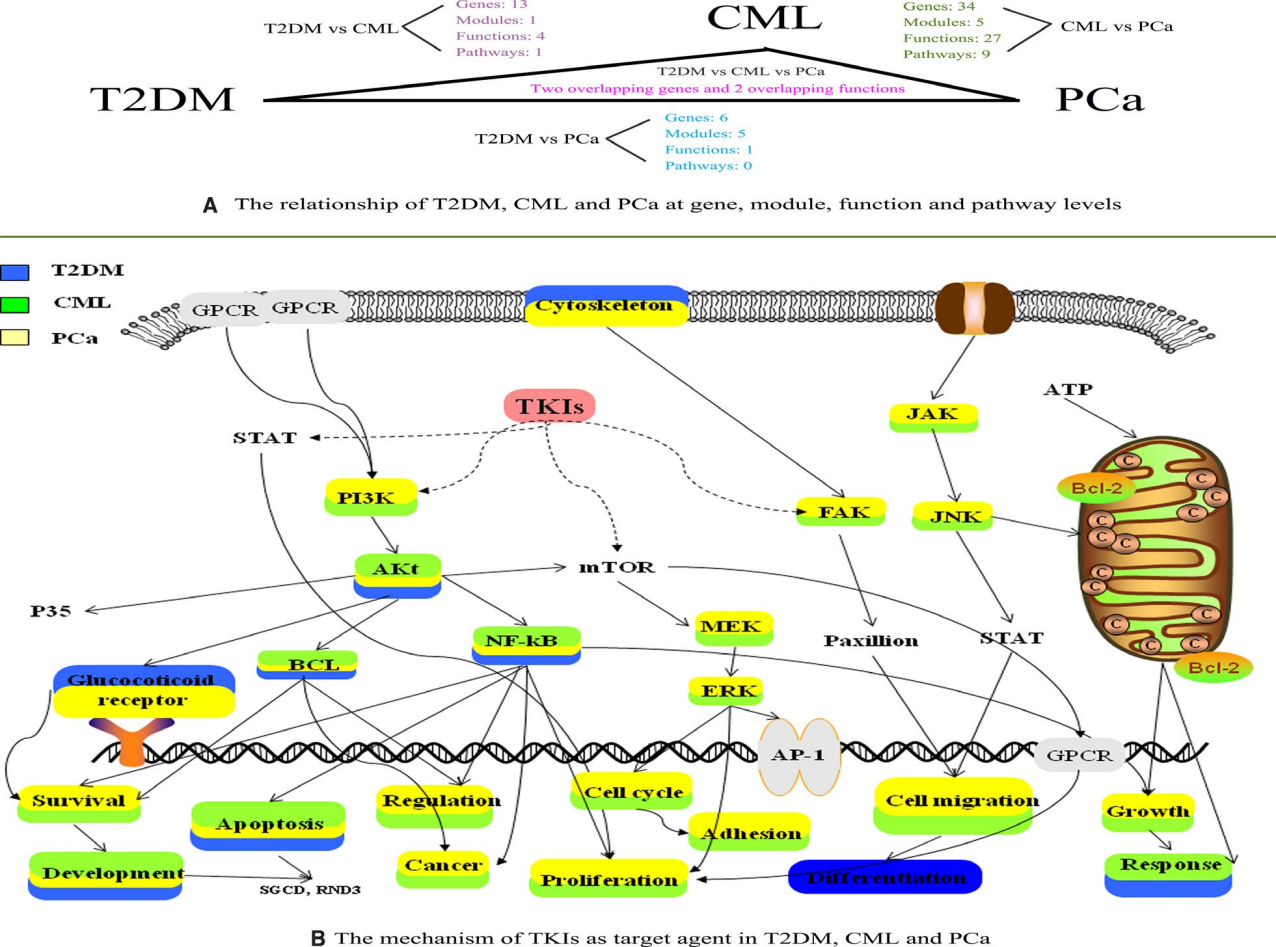
The relationship of the three diseases at gene, pathway, module, and function levels and the biological mechanism of T2DM, CML, and PCa. A, The comparison of genes, modules, functions, and pathways in T2DM, CML, and PCa. B, The biological mechanism for T2DM, CML, and PCa (pathways and processes related to T2DM, CML, and PCa are labeled with blue, green, and yellow, respectively. The solid line represents the pathways related to TKIs; the dotted line represents the direct targets related to TKIs)

Tyrosine kinase inhibitors (TKIs), such as imatinib and dasatinib, have been proven to be effective in the treatment of CML and PCa, and in most situations can improve the pre‐existing diabetes or lower the average blood glucose levels to a certain degree.^6^ Moreover, TKIs are commonly used in the treatment of both diabetes and cancers in clinical practice.^26^ Based on published literatures, TKIs, as a drug for cancer, were involved in apoptotic process.^27^ Several studies have found that TKIs can cause changes in blood glucose levels and insulin secretion during CML and PCa treatment.^11^, ^28^ Development processes were found in the main process classification of T2DM, CML, and PCa. In cancer development process, TKIs also have been demonstrated to play a role through receptor tyrosine kinases (RTKs) signaling pathways.^29^ Recent studies on molecular therapies have indicated that TKIs may involve cell growth, migration, invasion, development, adhesion, proliferation, apoptosis, and angiogenesis via Nuclear factor‐kB (NF‐κB), Focal adhesion kinase (FAK), Janus kinase1/2 (JAK1/2), ETK, C‐Jun N‐terminal kinase (JNK), Mitogen activated kinase‐like protein (MAPK), Extracellular regulated MAP kinase (ERK), Phosphatidylinositol‐4,5‐bisphosphate 3‐kinase catalytic subunit alpha‐Serine/threonine kinase 1 (PI3K‐Akt), and other cancer‐related signaling pathways (Figure 6B).^26^, ^30^, ^31^, ^32^, ^33^, ^34^, ^35^, ^36^, ^37^, ^38^, ^39^, ^40^

According to our findings, CML and PCa were the most closely related, with 34 overlapping genes, five overlapping modules, 27 overlapping biological functions and nine overlapping pathways. For example, development, cell proliferation, and cell adhesion processes were the main biological processes both in CML and PCa. Cancer, cell adhesion, and PI3K‐Akt signaling pathways were the common pathways in CML and PCa. Prior studies have revealed that NF‐κB, FAK, JAK1/2, ETK, JNK, MAPK, and PI3K‐Akt signaling pathways were involved in processes: cell growth, migration, invasion, angiogenesis, and apoptosis in CML and PCa (Figure 6B).^26^, ^30^, ^31^, ^32^, ^33^, ^34^, ^35^, ^36^, ^37^, ^38^, ^39^, ^40^ Liao et al^41^ discovered that PCa and CML were related to apoptosis by tyrosine kinases treating.

As for the relationship between T2DM and CML, 13 overlapping genes, one overlapping modules, four overlapping biological functions and one overlapping pathway (FoxO signaling pathway) were found. It has been shown that response to glucose process was related to CML.^6^, ^28^, ^42^

And T2DM and PCa were the least related pair in our study, with only six overlapping genes, five overlapping modules, and one overlapping biological function (endocrine pancreas development). Endocrine pancreas development has been associated with the risk of prostate cancer as well as insulin release from β cells in patients with T2DM.^43^, ^44^, ^45^

In summary, SGCD and RND3 were the main gene‐to‐gene relationship among T2DM, CML, and PCa; apoptosis, development, and transcription from RNA polymerase II promote processes were the main functional connections among T2DM, CML, and PCa by network enrichment analysis. There is a “scalene” relationship among T2DM, CML, and PCa at gene, pathway, biological process, and module levels: CML and PCa were the most closely related, the second were T2DM and PCa, and T2DM and PCa were the least related pair in our study (Figure 6A). Our study provides a new avenue for further studies on T2DM and cancers, which may promote the discovery and development of novel therapeutic and can be used to treat multiple diseases.

We also acknowledge some limitations of the present study. The last update of the database (Disease‐Connect website, https://disease-connect.org/) was on 31 December 2015, which may lead to incomplete data on disease‐related genes, and this needs to be addressed in our further studies.

## CONFLICT OF INTEREST

The authors declare that they have no competing financial interests.
