# Supplementary material for: Deciphering the scalene association among type‐2 diabetes mellitus, prostate cancer, and chronic myeloid leukemia via enrichment analysis of disease‐gene network
Source: Cancer Med. 2019 Apr 1;8(5):2268–77. doi: 10.1002/cam4.1845 (PMC6536925; doi:10.1002/cam4.1845)
Supplement: Supplementary file 1 [file CAM4-8-2268-s001.docx]

**Table S1 The Related genes in the Disease-connect**

| **Disease** | **Numbers** | **Disease-related genes** |
| --- | --- | --- |
| T2DM | 233 | 11-Sep, ABCC8, ABCG1, ACER3, ADAM10, ADAMTS9-AS2, ADCY5, AFTPH, AKT2, ALKBH6, ANAPC4, ANK1, ANKHD1-EIF4EBP3, AP1M1, AP3S2, ARAP1, ARFGEF1, ARHGEF1, ARL6IP5, ASAP1, ATP6V1E1, BAX, BLK, BLOC1S6, BNIP2, C15orf38-AP3S2, C2CD4A, C2CD4B, C6orf57, CABIN1, CAPN10, CCDC126, CDC123, CDKAL1, CDKN2B-AS1, CEL, CELP, CETN3, CGGBP1, CHCHD2P9, CHM, CLINT1, CLIP4, CMIP, CNN2, CRBN, CTBP1-AS2, DAZAP1, DCD, DHX15, DMRTA1, DNM1L, DPP8, DUSP9, DYM, EIF3EP3, EXOC6, FABP5P10, FAH, FAM13B, FAM19A1, FAM58A, FAT3, FITM2, FNDC3A, FNIP2, FOXO1, FSCN3, FTO, FYTTD1, GCC1, GCGR, GCK, GLIS3, GLO1, GNB4, GPD2, GPS2, GRK5, GRK6, GSAP, GSTM5P1, GTF2H1, HHEX, HIGD1A, HINT3, HLA-DQA2, HMG20A, HNF1A, HNF1A-AS1, HNF1B, HNF4A, HNRNPD, HNRNPKP3, HNRNPUL1, IDDM2, IGF2BP2, INS, IRS1, IRS2, ITPR3, JAZF1, KCNJ11, KCNK16, KCNK17, KCNQ1, KCNQ1OT1, KLF11, KLF14, KRT18P24, KRT18P48, LAMA1, LGR5, LMNA, LOC646736, MAEA, MAF, MAN2A1, MAPK8IP1, ME2, METTL25, MIR29A, MIR3660, MIR4432, MIR4480, MIR486, MIR5590, MIR5702, MTCO3P1, MTNR1B, NAA25, NARS, NDFIP2, NEUROD1, NIDDM2, NIDDM3, NIDDM4, NOTCH2, NYAP2, OASL, ORMDL3, OTUD5, PAM, PAX4, PCNXL2, PDLIM2, PDX1, PEPD, PHF5A, PNPLA8, POLK, PPIA, PPIGP1, PPP1R35, PRC1, PRICKLE2-AS1, PRKCSH, PSMA6, PSMB7, PSMD6, PTPRD, R3HDML, RAD50, RALY, RASA1, RASGRP1, RBMS1, RGS18, RND3, ROBO3, RPL12P33, RPL9P23, RPRD1A, RPS21P5, RPS27P20, RPS3P7, RPS6KA3, RPSAP52, RWDD1, SAP30L-AS1, SASH1, SF3A2, SF3B4, SGCD, SGCG, SH2B3, SLC25A28, SLC2A2, SLC2A4, SLC30A8, SLC39A6, SNORA70F, SON, SPRY2, SRR, ST6GAL1, SVEP1, SYN2, TAF10, TAOK3, TCF4, TCF7L2, TFRC, THADA, TIMM50, TMED9, TMEM163, TMEM167B, TMEM45B, TP53INP1, TRAPPC11, TSPAN8, U2AF2, UCP3, UGP2, UMOD, VDAC1P5, VPS26A, VPS8, WDR37, WFS1, XRCC5, YY1AP1, ZBED3-AS1, ZC3H13, ZCCHC6, ZFAND3, ZFAND6, ZMYM6, ZNF207, ZNF548, ZNF700, ZNF800 |
| CML | 735 | 10-Sep, 5-Sep, ABCA12, ABCC4, ABL1, ABLIM1, ACOT13, ACOT4, ADAMTS1, ADAMTS5, ADI1, AFF3, AGR3, AIF1, AKR1C2, AKR7A2, ALCAM, ALDH1A1, ALDH1A2, ALDH1L2, ALDH7A1, AMIGO2, ANKRD22, ANKRD29, ANXA2, ANXA5, AP1M2, APBB1IP, APP, ARCN1, ARHGAP30, ARHGAP4, ARHGEF12, ARHGEF9, ARL4A, ARL6, ARL6IP5, ARMCX2, ARNT2, ASAP2, ASNSD1, ATF5, ATP5L, ATP8B1, B3GALNT1, BACE2, BASP1, BBS4, BBS9, BCAN, BCAT1, BCHE, BCL11A, BCL2A1, BCL6, BCR, BEX1, BEX4, BMP1, BNIP3, BRAF, BTG3, BTK, BTNL9, C10orf128, C10orf76, C15orf48, C16orf54, C1orf21, C1orf228, C1orf61, C20orf196, C2CD2L, C4orf33, C6orf89, C7orf13, C8orf46, CADM1, CALD1, CALR, CAMK2D, CAMSAP2, CAND2, CAP2, CAPN2, CAPN3, CASP4, CAST, CAV2, CBFB, CCDC26, CCDC28B, CCHCR1, CCND2, CCNG2, CD200, CD302, CD36, CD40, CD55, CDCA2, CDH1, CDH11, CDH2, CDH6, CDKN2A, CDKN2B, CDON, CEBPA, CEBPG, CELSR1, CEP57, CEP57L1, CFHR1, CHCHD10, CHI3L1, CHMP4C, CHST15, CHST6, CHST7, CHTOP, CISH, CIZ1, CKAP4, CLDN1, CLSPN, CLU, CMTM8, CNN3, COL12A1, COL1A1, COL1A2, COL5A1, COL5A2, COL6A2, COLGALT2, COMMD4, COPS7A, COX7A2L, CR1L, CREG1, CRKL, CSPG4, CTBP2, CTCFL, CTGF, CTHRC1, CTSA, CTSO, CX3CR1, CXADR, CXCR4, CXorf48, CXorf61, CYAT1, CYR61, DBF4B, DCAF12L1, DCTN3, DDX26B, DESI1, DGCR2, DHDH, DIP2C, DIRC2, DLK1, DMRT1, DMXL1, DNAJC6, DOCK5, DPP4, DPP7, DPYD, DPYSL3, DSCR8, DSEL, DSP, DUSP4, DYNC1LI2, DZIP1, EDNRB, EFEMP1, EFEMP2, EFR3A, EGFR, EHD1, EHHADH, EIF1AY, ELF4, ELMO2, ELOVL7, EML1, EMP1, ENPP4, EPB41, EPCAM, EPDR1, EPG5, EPHX1, EPHX2, EPM2AIP1, ERBB3, ERI1, ERN1, ESRRB, ETFA, EXPH5, FAF1, FAM117A, FAM132B, FAM184A, FAM210B, FAM45A, FAM57A, FAM65C, FAM78A, FAM83A, FAS, FBN1, FEM1C, FERMT3, FGF13, FGFR1OP, FOSL2, FRAS1, FSTL1, FZD6, FZD8, G0S2, GAB2, GABARAPL2, GAGE12D, GAGE12F, GAGE8, GALM, GALNT3, GALNT5, GAS1, GAS2L3, GATA2, GBAP1, GBP1, GBP3, GCAT, GDF15, GFPT2, GFRA1, GJA1, GJC1, GLIPR1, GLRB, GLRX2, GLT8D2, GMFB, GNA15, GNAI1, GNB2, GNB5, GPM6B, GPNMB, GPR177, GPR19, GPR56, GPX1, GPX8, GREM1, GSAP, GSTT1, GTSF1, GYPB, GYPE, GZMA, H1F0, H2BFS, HAS2, HBA1, HBA2, HBB, HBE1, HBG2, HCG11, HEXB, HGF, HHEX, HIBADH, HIST1H1C, HIST1H2AE, HIST1H2AM, HIST1H2BD, HIST1H2BE, HIST1H4I, HIVEP2, HLA-A, HLA-B, HLA-C, HLA-DQB1, HLA-F, HMCN1, HMG20B, HMHA1, HOOK1, HOXA10, HOXB2, HOXB9, HRH1, HS2ST1, HS3ST3B1, HSD3B7, HTRA1, HTRA3, ICAM1, IFFO1, IFI27, IFIT5, IFITM1, IFNG, IFT43, IGFBP6, IGFBP7, IGJ, IL17RB, INHBA, INHBE, INPP4B, INPP5F, IPW, IQCB1, IQGAP2, IRF4, IRS1, ITGA4, ITGB3, ITGB8, ITK, JAK2, KCNE4, KCNH2, KCNK1, KCTD12, KDM2A, KIAA1211, KIAA1462, KIAA1644, KIAA1804, KIF3B, KIRREL, KL, KLHL4, KRT34, KRT80, L1CAM, LAMP1, LCP1, LDHB, LEPR, LGALS3BP, LGMN, LIN28B, LINC00328, LINC00969, LMCD1, LMO2, LOC100287497, LOC100506014, LOC285628, LOC389831, LOC729810, LOX, LOXL2, LPHN2, LPP, LRRC16A, LRRCC1, LRRK2, LXN, LY75, LYL1, LYN, MAF, MAGEA12, MAGEA3, MAGEA5, MAGEA6, MAGEB2, MAGED1, MAL2, MAN1A1, MAN2A2, MAP3K13, MAP3K4, MAP9, MAPK1, MARCKS, MAST4, MATN2, MCFD2, MCM7, MEF2C, METTL7A, MFAP5, MGAT4A, MGAT4B, MGC39584, MGST2, MIPOL1, MIR100HG, MLANA, MLKL, MLLT11, MNDA, MOCOS, MOG, MORC4, MPL, MPP1, MPP5, MRAP2, MRAS, MS4A3, MSRB3, MT1E, MTAP, MVP, MYBL1, MYH11, MYO10, MYO1B, MYO1F, MYO1G, MYO5A, MYO5C, MYO6, MYOF, NAGS, NAMPT, NBL1, NCALD, NDUFB5, NEDD9, NEFL, NET1, NETO2, NFE2, NFIB, NFKBIA, NGFRAP1, NHS, NINJ2, NLRP2, NME1, NMU, NOS3, NOSTRIN, NOV, NPHP3, NR2F6, NRIP3, NRP2, NT5E, NTN4, NUP62CL, NUPR1, NVL, NXN, OAS1, OCIAD2, OGFRL1, OR51B4, OSBPL1A, PABPC4L, PAGE1, PALLD, PALM2-AKAP2, PAM, PAN3, PAPSS2, PARPBP, PARVA, PAWR, PCDH18, PCDHA1, PCNX, PCNXL2, PDAP1, PDGFC, PDGFRB, PDLIM1, PDLIM2, PEG10, PEPD, PFN2, PGM1, PHF11, PHF19, PHLDB2, PI4KA, PID1, PIGS, PIM1, PIP, PKMYT1, PKP3, PLA2G12A, PLAT, PLAUR, PLEKHH1, PLGRKT, PLK2, PLOD2, PLP1, PLS3, PLXDC1, PMEPA1, PMP2, PMP22, PODXL, POU2AF1, PPAP2B, PPARG, PPP1R14C, PPP2R3A, PRAME, PRKAB1, PRKAR2B, PRKCDBP, PRODH, PROS1, PRRX1, PRSS23, PSD4, PSEN2, PSG5, PTGER3, PTP4A1, PTP4A3, PTPLA, PTPN14, PTPN21, PTPN3, PTPRF, PTPRG, PTPRK, PTPRN2, PTRF, PYCR2, RAB13, RAB24, RAB27A, RAB34, RAB38, RAB7A, RABGAP1, RAD51, RAGE, RARRES2, RASSF5, RBBP8, RBFOX2, RBMS1, RBMS3, RDH10, REEP5, RELN, RFC2, RHOBTB3, RIMS2, RIN2, RNASE4, RNF125, RNF128, RNF13, ROBO1, ROD1, ROPN1B, RPL31, RPL34P31, RPL36A, RPS3A, RPS4Y1, RPS6KA2, RPS6KA3, RRAS, S100A10, S100A13, S100B, SAGE1, SCARB2, SCUBE2, SDC2, SELM, SEMA4D, SERINC5, SERPINB2, SERPINB3, SERPINE1, SERPINF1, SETD8, SFRP2, SGCD, SGPL1, SH2B3, SH3KBP1, SIGMAR1, SLC10A4, SLC10A7, SLC12A8, SLC13A3, SLC1A3, SLC25A21, SLC2A10, SLC35D2, SLC39A8, SLC44A4, SLC45A2, SLC6A15, SLC8A1, SLC9A1, SLFN5, SMAD2, SMAP2, SNCA, SNX21, SOWAHC, SOX10, SOX4, SOX9, SPINT2, SPRED1, SRGN, SRP72, ST14, ST3GAL6, STAR, STK3, STMN1, STON1, STON2, STRBP, STT3B, STXBP2, STXBP6, SUSD1, SVIL, SYNGR3, SYTL2, TAGLN, TAGLN2, TANC1, TBC1D16, TBP, TCEAL1, TCF7L2, TDRG1, TERC, TERT, TGFB1I1, TGFBR2, TGIF1, THBS1, THY1, TIMP2, TIPARP, TLE1, TLR4, TLR8-AS1, TM4SF1, TM6SF1, TMBIM1, TMCC3, TMEFF2, TMEM14A, TMEM158, TMEM230, TMEM255A, TMEM30B, TMEM47, TMOD1, TMOD3, TNFRSF11B, TNFRSF19, TNFSF10, TOMM20, TP53, TPBG, TPD52L1, TPRG1, TRAF3IP3, TRAFD1, TRIM21, TRIM39-RPP21, TRNP1, TSPAN12, TSPAN13, TSPAN6, TTC13, TTLL3, TUBB6, TUFMP1, TUSC2, TVP23B, UBE2C, UBR2, UBXN8, UCHL1, UHRF2, UQCRH, UST, VAMP8, VCAN, VDAC2, VPS13D, WDR82, WNT5A, WRN, WWTR1, XAGE1B, XIST, YAP1, YIF1B, YOD1, ZAK, ZBED2, ZC3HAV1L, ZFPM2, ZHX2, ZIC2, ZNF137P, ZNF280B, ZNF382, ZNF595, ZNF667-AS1, ZNF711, ZNF827 |
| PCa | 511 | ABCA3, ACIN1, ACP5, ACSS3, ACTA2, ACTG2, ADIRF, AFM, AGAP7, AGTR1, AKIRIN2, ALB, ALCAM, ALG1L, AMACR, AMBP, AMD1, AMIGO2, ANKRD28, ANKRD62, AOX1, APBB3, APCS, AQP8, AR, ARHGEF3, ARMC2, ASCL2, ASS1P11, ATF3, ATP8B4, ATP9B, AUP1, AVPR1B, BAG2, BAIAP2L1, BCL6, BIK, BMP4, BNC1, BOD1, BRCA2, C15orf37, C18orf25, C1orf116, C1orf52, C2orf43, CA14, CALM2P1, CAPG, CBX2, CBX8, CCDC28B, CCHCR1, CCL2, CCND2, CCNH, CCT4P2, CD44, CD74, CD82, CD99, CDC42EP5, CDC42SE2, CDCP1, CDH1, CDK12, CDK7, CDKL3, CEBPG, CEL, CELA2A, CELA2B, CENPVP3, CEP152, CEP85, CHEK2, CHM, CHMP5, CHN2, CIPC, CKMT1A, CLDN11, CLDN7, CMKLR1, CMTM3, CNN1, CNTNAP5, COL14A1, COL1A2, COL4A3BP, COL5A1, COL6A1, COL6A3, CPED1, CRISP3, CROCCP2, CRP, CSAD, CTBP2, CTBS, CTRC, CTRL, CTSH, CTSO, CUZD1, CWC27, CYR61, DCAF4L1, DCN, DDX19B, DENND5A, DHRS3, DIO2, DNAH12, DPEP1, DPF1, DPP10, DST, DYNC1I1, EBF2, EBPL, EEFSEC, EFEMP2, EFNB1, EFS, EGF, EGR2, EGR3, EHBP1, ELAC2, ENSP00000295220, ENSP00000381049, EOGT, EPB41L2, EPCAM, EPS8, ERP27, EYA2, F11R, F3, FAM110B, FAM35A, FAM60A, FAM60DP, FARP2, FBLN1, FERMT2, FGA, FGF10, FGFBP1, FGFR2, FGFR3, FGG, FGL1, FICD, FKBP1B, FLJ40194, FLNC, FMOD, FNBP1L, FOLR3, FOS, FOSB, FOXP4, FSHR, FXYD1, FXYD3, GALC, GALNT12, GAS1, GATA5, GBP1, GCOM1, GGCX, GGT2, GJB3, GLULP5, GNA11, GNAL, GNMT, GP2, GPHA2, GPR153, GPRASP1, GPRC5C, GREM2, GRHL1, GSTA2, GSTA4, GSTM1, GSTM2, GSTM5, GUCA1C, GYG2, HBEGF, HEXIM2, HHIP, HIGD1AP18, HIP1, HJURP, HLA-DPB1, HMGN2P32, HNF1B, HOXB7, HOXC6, HPC10, HPC10, HPC15, HPC4, HPC5, HPC6, HPC7, HPC9, HPCQTL19, HPCX2, HPSE2, HRAS, HS3ST3A1, HSD17B7, IFIT1, IGF1, IGFBP5, IGFBPL1, IL22RA1, INO80D, IPO9, IRF2, IRX2, IRX4, ITGA6, ITGB8, ITM2C, KCNK5, KCNN3, KCNN4, KCNS3, KIAA1244, KLF6, KLK1, KLK2, KLK3, KNTC1, KRT19, KRT6A, KRT78, LAMA3, LAMA4, LAMB3, LAMC2, LDLRAP1, LILRA3, LMOD1, LMTK2, LOC284581, LONP1, LPAR3, LPGAT1, LRPPRC, LRRC32, LRRC40, LRRIQ1, LRRN4CL, LSAMP, LTF, LYPD3, MAD1L1, MAFG, MAFG-AS1, MAMDC2, MAPK13, MARVELD2, MDM4, MED13L, MEIS2, MET, METAP1, MFAP4, MIR3164, MIR4686, MIR4752, MIR4795, MLPH, MMP28, MMP7, MOBKL2B, MRPL43, MRPS21, MSMB, MSR1, MX1, MXI1, MXRA7, MXRA8, MYC, MYEOV, MYH11, MYLK, MYO6, MYOCD,,MYOF, NAGPA, NBEAL2, NFATC3, NFX1, NKX3-1, NLK, NOMO3, NOTCH4, NPTX2, NR1H4, NREP, NUDT10, NUDT11, NUP188, ONECUT1, OR4D5, OR7E108P, OR8D4, OR8S21P, OTX1, P2RX4, PAGE4, PCAT1, PDE11A, PDIA2, PDLIM5, PGK1P1, PGM5-AS1, PHIP, PHYKPL, PI4KB, PKIA, PLCH2, PNLIPRP1, PNLIPRP2, POU5F1B, PPA2, PPAP2B, PPATP1, PPIAP6, PPP1R13L, PPP1R14A, PPP2CA, PPP4R1, PPT1, PRDM2, PREPL, PRICKLE2, PRKAR2B, PRKCSH, PRNP, PRPH, PRRX2, PRSS16, PSG5, PSMG1, PTEN, PTGFRN, PYGL, RAB12, RAB28, RAD23B, RBBP8NL, RBPJL, RCN1, RCN3, REG3G, RFX6, RGS17, RIOK2, RNASE9, RNASEL, RNASEN, RND3, RNFT2, RNU6-66P, RNU7-89P, RPL12P7, RPL19P16, RPL21, RPL22L1, RPL23AP61, RPL27P5, RPL6, RPL6P14, RPL7P41, RPS14P3, RPS25P10, RPS26P30, RRAGD, RSL24D1, RWDD2A, RWDD2B, SALL3, SCGB3A1, SCTR, SDC1, SDCBP, SDK2, SEC14L2, SEL1L, SEMA5A, SEMA6D, SENP5, SERPINA5, SERPINA6, SERPINB5, SERPINB6, SERPINH1, SERPINI2, SFMBT1, SFRP2, SFRP5, SGCD, SGCG, SHROOM2, SIDT1, SKIL, SLC10A2, SLC14A1, SLC16A5, SLC1A2, SLC22A1, SLC22A2, SLC22A3, SLC22A4, SLC38A3, SLC39A5, SLC4A4, SMARCA1, SMOC2, SNED1, SOX9, SPINT2, SPON1, SPRR1A, SQRDL, SRD5A1, SRRM1P1, SSX2IP, ST3GAL5, ST6GALNAC1, STC2, STK11, SYCN, SYK, TBC1D19, TBX5, TC2N, TCF21, TDG, TERT, TET2, TFF3, THADA, THRB, THY1, TJP3, TMBIM1, TMED2, TMEM168, TMEM181, TMEM200B, TMEM260, TMEM30B, TMEM40, TMEM97, TNRC6B, TNS1, TOX2, TOX4, TP63, TRIM13, TRIM74, TRIM8, TRPV5, TSN, TSNARE1, TTC7A, TTRAP, TUBA1C, UCP2, UNG, UPF2, VAMP8, VCAM1, VEPH1, VPS53, VSNL1, WDR34, WFDC1, WNK2, WNT7A, YWHAG, ZBED2, ZBTB38, ZC4H2, ZDHHC14, ZFHX3, ZGPAT, ZNF281, ZNF615, ZNF652, ZNF655, ZNF672, ZNF804A, ZNF827, ZNHIT6 |
