# Supplementary material for: Deciphering the scalene association among type‐2 diabetes mellitus, prostate cancer, and chronic myeloid leukemia via enrichment analysis of disease‐gene network
Source: Cancer Med. 2019 Apr 1;8(5):2268–77. doi: 10.1002/cam4.1845 (PMC6536925; doi:10.1002/cam4.1845)
Supplement: Supplementary file 2 [file CAM4-8-2268-s002.docx]

**Table S2 The result of enrichment analysis for T2DM-related genes**

|  | **Category** | **Term** | **P-Value** | **Genes List** |
| --- | --- | --- | --- | --- |
| 1 | GOTERM_BP_DIRECT | GO:0042593~glucose homeostasis | 1.17E-09 | HNF1A, HNF4A, SLC2A4, GCK, INS, WFS1, MTNR1B, NEUROD1, PDX1, SLC30A8, TCF7L2, IRS1, GCGR |
| 2 | GOTERM_BP_DIRECT | GO:0050796~regulation of insulin secretion | 5.05E-07 | HNF1A, HNF4A, GCK, SLC2A2, MTNR1B, NEUROD1, ITPR3, ABCC8, KCNJ11 |
| 3 | GOTERM_BP_DIRECT | GO:0009749~response to glucose | 1.42E-05 | IRS2, HNF1B, HNF1A, HNF4A, SLC2A2, NEUROD1, SLC30A8, TCF7L2 |
| 4 | GOTERM_BP_DIRECT | GO:0045725~positive regulation of glycogen biosynthetic process | 1.59E-05 | IRS2, GCK, INS, IRS1, AKT2 |
| 5 | GOTERM_BP_DIRECT | GO:2000675~negative regulation of type B pancreatic cell apoptotic process | 2.37E-05 | WFS1, NEUROD1, PDX1, TCF7L2 |
| 6 | GOTERM_BP_DIRECT | GO:0032024~positive regulation of insulin secretion | 8.27E-05 | IRS2, CAPN10, GCK, BLK, SLC30A8, TCF7L2 |
| 7 | GOTERM_BP_DIRECT | GO:0031018~endocrine pancreas development | 1.85E-04 | HNF1B, HNF1A, FOXO1, NEUROD1, PAX4 |
| 8 | GOTERM_BP_DIRECT | GO:0046326~positive regulation of glucose import | 2.82E-04 | IRS2, CAPN10, INS, IRS1, AKT2 |
| 9 | GOTERM_BP_DIRECT | GO:0032869~cellular response to insulin stimulus | 3.66E-04 | IRS2, CAPN10, GCK, SLC2A4, FOXO1, IRS1, AKT2 |
| 10 | GOTERM_BP_DIRECT | GO:0030073~insulin secretion | 4.10E-04 | HNF1B, HNF1A, NEUROD1, PDX1, SLC30A8 |
| 11 | GOTERM_BP_DIRECT | GO:0042493~response to drug | 0.001495 | XRCC5, PAM, HNF1B, HNF4A, BAX, SRR, NEUROD1, PAX4, PDX1, GRK5, ABCC8, KCNJ11 |
| 12 | GOTERM_BP_DIRECT | GO:0008286~insulin receptor signaling pathway | 0.001592 | IRS2, INS, ATP6V1E1, FOXO1, IRS1, AKT2 |
| 13 | GOTERM_BP_DIRECT | GO:0000165~MAPK cascade | 0.002055 | IRS2, PSMB7, PSMA6, INS, RASGRP1, TAOK3, PSMD6, DUSP9, IRS1, RASA1 |
| 14 | GOTERM_BP_DIRECT | GO:0032000~positive regulation of fatty acid beta-oxidation | 0.003917 | IRS2, IRS1, AKT2 |
| 15 | GOTERM_BP_DIRECT | GO:0010907~positive regulation of glucose metabolic process | 0.003917 | IRS2, IRS1, AKT2 |
| 16 | GOTERM_BP_DIRECT | GO:0000122~negative regulation of transcription from RNA polymerase II promoter | 0.005508 | MAF, GLIS3, HNF1B, HNF1A, WFS1, KLF11, FOXO1, HMG20A, PAX4, PDX1, TCF7L2, GPS2, FNIP2, HHEX, CGGBP1, JAZF1, TCF4 |
| 17 | GOTERM_BP_DIRECT | GO:0015758~glucose transport | 0.005683 | GCK, SLC2A4, INS, SLC2A2 |
| 18 | GOTERM_BP_DIRECT | GO:0006006~glucose metabolic process | 0.006107 | IRS2, INS, PDX1, KCNJ11, AKT2 |
| 19 | GOTERM_BP_DIRECT | GO:0046676~negative regulation of insulin secretion | 0.006676 | MTNR1B, ABCC8, IRS1, KCNJ11 |
| 20 | GOTERM_BP_DIRECT | GO:0045597~positive regulation of cell differentiation | 0.007765 | RPS6KA3, INS, NEUROD1, PAX4 |
| 21 | GOTERM_BP_DIRECT | GO:0043547~positive regulation of GTPase activity | 0.00788 | IRS2, ARHGEF1, DNM1L, ASAP1, RGS18, ARFGEF1, GCGR, IRS1, FAM13B, BNIP2, RASGRP1, CHM, RASA1, ARAP1 |
| 22 | GOTERM_BP_DIRECT | GO:0060047~heart contraction | 0.00956 | DNM1L, SGCG, SGCD |
| 23 | GOTERM_BP_DIRECT | GO:0000398~mRNA splicing, via spliceosome | 0.010139 | RALY, HNRNPUL1, U2AF2, HNRNPD, DHX15, PHF5A, SF3A2, SF3B4 |
| 24 | GOTERM_BP_DIRECT | GO:0048839~inner ear development | 0.010926 | MAF, NEUROD1, KCNQ1, LGR5 |
| 25 | GOTERM_BP_DIRECT | GO:0043066~negative regulation of apoptotic process | 0.011943 | ZFAND6, SPRY2, NOTCH2, RPS6KA3, SON, BNIP2, BLK, FOXO1, GLO1, PAX4, GRK5, HIGD1A |
| 26 | GOTERM_BP_DIRECT | GO:0006521~regulation of cellular amino acid metabolic process | 0.017326 | PSMB7, PSMA6, INS, PSMD6 |
| 27 | GOTERM_BP_DIRECT | GO:0001889~liver development | 0.019126 | MAN2A1, HNF1A, HNRNPD, PDX1, PRKCSH |
| 28 | GOTERM_BP_DIRECT | GO:0061017~hepatoblast differentiation | 0.021331 | HHEX, HNF1B |
| 29 | GOTERM_BP_DIRECT | GO:0035565~regulation of pronephros size | 0.021331 | HNF1B, HNF1A |
| 30 | GOTERM_BP_DIRECT | GO:0016192~vesicle-mediated transport | 0.022843 | TRAPPC11, C15ORF38-AP3S2, AP1M1, AP3S2, MAPK8IP1, CLINT1 |
| 31 | GOTERM_BP_DIRECT | GO:0031016~pancreas development | 0.024956 | HHEX, WFS1, TCF7L2 |
| 32 | GOTERM_BP_DIRECT | GO:0046688~response to copper ion | 0.027036 | PAM, TFRC, BAX |
| 33 | GOTERM_BP_DIRECT | GO:0001836~release of cytochrome c from mitochondria | 0.027036 | DNM1L, BAX, TIMM50 |
| 34 | GOTERM_BP_DIRECT | GO:0007010~cytoskeleton organization | 0.029802 | ANK1, MAEA, ZMYM6, BLK, FITM2, CNN2 |
| 35 | GOTERM_BP_DIRECT | GO:0051594~detection of glucose | 0.031826 | GCK, PDX1 |
| 36 | GOTERM_BP_DIRECT | GO:0005978~glycogen biosynthetic process | 0.033672 | GCK, UGP2, AKT2 |
| 37 | GOTERM_BP_DIRECT | GO:0051436~negative regulation of ubiquitin-protein ligase activity involved in mitotic cell cycle | 0.040762 | PSMB7, PSMA6, ANAPC4, PSMD6 |
| 38 | GOTERM_BP_DIRECT | GO:0071805~potassium ion transmembrane transport | 0.041319 | KCNK17, KCNK16, ABCC8, KCNQ1, KCNJ11 |
| 39 | GOTERM_BP_DIRECT | GO:0010748~negative regulation of plasma membrane long-chain fatty acid transport | 0.042209 | IRS2, AKT2 |
| 40 | GOTERM_BP_DIRECT | GO:0002029~desensitization of G-protein coupled receptor protein signaling pathway | 0.042209 | GRK6, GRK5 |
| 41 | GOTERM_BP_DIRECT | GO:0010638~positive regulation of organelle organization | 0.042209 | ANK1, TMED9 |
| 42 | GOTERM_BP_DIRECT | GO:0045444~fat cell differentiation | 0.045182 | FOXO1, GRK5, TCF7L2, AKT2 |
| 43 | GOTERM_BP_DIRECT | GO:0051437~positive regulation of ubiquitin-protein ligase activity involved in regulation of mitotic cell cycle transition | 0.048255 | PSMB7, PSMA6, ANAPC4, PSMD6 |
| 1 | KEGG_PATHWAY | hsa04950:Maturity onset diabetes of the young | 1.52E-11 | HHEX, HNF1B, HNF1A, HNF4A, GCK, INS, SLC2A2, NEUROD1, PAX4, PDX1 |
| 2 | KEGG_PATHWAY | hsa04930:Type II diabetes mellitus | 1.25E-07 | IRS2, GCK, SLC2A4, INS, SLC2A2, PDX1, ABCC8, IRS1, KCNJ11 |
| 3 | KEGG_PATHWAY | hsa04911:Insulin secretion | 9.43E-05 | GCK, INS, ADCY5, SLC2A2, PDX1, ITPR3, ABCC8, KCNJ11 |
| 4 | KEGG_PATHWAY | hsa04931:Insulin resistance | 4.21E-04 | IRS2, RPS6KA3, SLC2A4, INS, SLC2A2, FOXO1, IRS1, AKT2 |
| 5 | KEGG_PATHWAY | hsa04152:AMPK signaling pathway | 0.004463 | IRS2, HNF4A, SLC2A4, INS, FOXO1, IRS1, AKT2 |
| 6 | KEGG_PATHWAY | hsa04923:Regulation of lipolysis in adipocytes | 0.005448 | IRS2, INS, ADCY5, IRS1, AKT2 |
| 7 | KEGG_PATHWAY | hsa04910:Insulin signaling pathway | 0.008075 | IRS2, GCK, SLC2A4, INS, FOXO1, IRS1, AKT2 |
| 8 | KEGG_PATHWAY | hsa04922:Glucagon signaling pathway | 0.008366 | GCK, SLC2A2, FOXO1, ITPR3, GCGR, AKT2 |
| 9 | KEGG_PATHWAY | hsa04914:Progesterone-mediated oocyte maturation | 0.024550 | RPS6KA3, INS, ADCY5, ANAPC4, AKT2 |
| 10 | KEGG_PATHWAY | hsa04068:FoxO signaling pathway | 0.027682 | IRS2, SLC2A4, INS, FOXO1, IRS1, AKT2 |
| 11 | KEGG_PATHWAY | hsa04150:mTOR signaling pathway | 0.037266 | RPS6KA3, INS, IRS1, AKT2 |
| 12 | KEGG_PATHWAY | hsa04114:Oocyte meiosis | 0.049825 | RPS6KA3, INS, ADCY5, ANAPC4, ITPR3 |
