# Supplementary material for: Deciphering the scalene association among type‐2 diabetes mellitus, prostate cancer, and chronic myeloid leukemia via enrichment analysis of disease‐gene network
Source: Cancer Med. 2019 Apr 1;8(5):2268–77. doi: 10.1002/cam4.1845 (PMC6536925; doi:10.1002/cam4.1845)
Supplement: Supplementary file 3 [file CAM4-8-2268-s003.docx]

**Table S3 The result of enrichment analysis for CML-related genes**

|  | **Category** | **Term** | **P-value** | **Genes List** |
| --- | --- | --- | --- | --- |
| 1 | GOTERM_BP_DIRECT | GO:0007155~cell adhesion | 2.96E-09 | NRP2, IGFBP7, NINJ2, BCAN, NEDD9, L1CAM, PCDHA1, ITGB3, CDH2, MOG, CDH6, ALCAM, DGCR2, LGALS3BP, APP, KIAA1462, ITGB8, CTGF, ROBO1, COL6A2, COL12A1, THBS1, GPNMB, LOXL2, CYR61, ICAM1, PTPRK, PTPRF, LPP, PODXL, ITGA4, TPBG, COL5A1, PCDH18, THY1, RND3, CD36, CDON, CX3CR1, VCAN, RELN, COL1A1, TGFB1I1, SEMA4D, PARVA, CDH11 |
| 2 | GOTERM_BP_DIRECT | GO:0007596~blood coagulation | 3.76E-07 | PLAT, NFE2, LYN, ENPP4, HMG20B, ITGB3, ANXA5, PLAUR, PRKAR2B, GATA2, CD36, COL1A2, SH2B3, PDGFC, ZFPM2, JAK2, COL1A1, HBG2, EHD1, HBE1, PAPSS2, HBB, PROS1, RAB27A |
| 3 | GOTERM_BP_DIRECT | GO:0043065~positive regulation of apoptotic process | 6.79E-07 | ZAK, CLU, SNCA, SOX4, BNIP3, PAWR, ARHGAP4, ALDH1A2, CDKN2A, BCL6, FAS, ARL6IP5, NET1, GZMA, BCL2A1, TP53, ARHGEF9, ARHGEF12, ANXA5, STK3, TNFSF10, S100B, NUPR1, SFRP2, RPS6KA2, MLLT11, PSEN2, MNDA, PDGFRB, FAF1, ABL1, SLC9A1 |
| 4 | GOTERM_BP_DIRECT | GO:0030199~collagen fibril organization | 9.72E-06 | SFRP2, COL1A2, COL12A1, COL1A1, LOX, GREM1, LOXL2, COL5A2, COL5A1, ANXA2 |
| 5 | GOTERM_BP_DIRECT | GO:0008284~positive regulation of cell proliferation | 1.00E-05 | NAMPT, DBF4B, CLU, ARNT2, SOX4, GREM1, SOX9, CALR, EPCAM, ALDH1A2, EDNRB, AKR1C2, GAB2, CTGF, IFNG, PDGFC, YAP1, THBS1, DPP4, EGFR, CHTOP, PRAME, LYN, TGFBR2, EFEMP1, HGF, WWTR1, IRS1, S100A13, MAPK1, CRKL, S100B, SFRP2, CCND2, FGFR1OP, PDGFRB, HAS2, JAK2, SERPINB3 |
| 6 | GOTERM_BP_DIRECT | GO:0060333~interferon-gamma-mediated signaling pathway | 1.04E-05 | HLA-DQB1, ICAM1, IFNG, HLA-A, CAMK2D, OAS1, HLA-C, JAK2, HLA-B, IRF4, TRIM21, GBP1, HLA-F |
| 7 | GOTERM_BP_DIRECT | GO:0030198~extracellular matrix organization | 1.29E-05 | ICAM1, FBN1, BCAN, CDH1, ITGA4, ITGB3, SOX9, COL5A2, COL5A1, NPHP3, TNFRSF11B, APP, ITGB8, SERPINE1, COL1A2, COL6A2, VCAN, COL1A1, LOX, THBS1, MFAP5, CYR61 |
| 8 | GOTERM_BP_DIRECT | GO:1902042~negative regulation of extrinsic apoptotic signaling pathway via death domain receptors | 2.07E-05 | ICAM1, GPX1, TNFSF10, SFRP2, SERPINE1, NOS3, TMBIM1, FAS, HGF |
| 9 | GOTERM_BP_DIRECT | GO:0010628~positive regulation of gene expression | 3.25E-05 | MEF2C, STAR, ERBB3, CTCFL, GJA1, TLR4, PAWR, CALR, RIMS2, ALDH1A2, ITGB8, CTGF, IFNG, PIP, RAB27A, PID1, PLP1, PTGER3, BRAF, EPHX2, PRKAB1, TP53, TLE1, INHBA, RPS6KA2 |
| 10 | GOTERM_BP_DIRECT | GO:0042493~response to drug | 5.30E-05 | PAM, STAR, PPARG, SNCA, CDH1, CALR, TIMP2, TNFRSF11B, SLC1A3, MCM7, BCHE, IFNG, FAS, LOX, THBS1, ICAM1, SLC8A1, MYO6, PTGER3, LYN, TGFBR2, RAD51, INHBA, SFRP2, TGIF1, ABCC4, COL1A1, SRP72, ABL1, SLC9A1 |
| 11 | GOTERM_BP_DIRECT | GO:0016477~cell migration | 6.67E-05 | CTHRC1, PTPRK, PTPRF, PODXL, CSPG4, ITGB3, CDH2, PALLD, COL5A1, SDC2, ELMO2, CTGF, SH3KBP1, PDGFRB, JAK2, THBS1, ABL1, LCP1, SLC9A1 |
| 12 | GOTERM_BP_DIRECT | GO:0008285~negative regulation of cell proliferation | 1.39E-04 | IFITM1, IGFBP7, IGFBP6, HIST1H2AE, SOX4, TIMP2, ALDH1A2, CDKN2A, CDKN2B, BCHE, BCL6, NOS3, GPNMB, CEBPA, PTPRK, CTBP2, LYN, TP53, PTPN14, SMAD2, STK3, ATF5, INHBA, RASSF5, NME1, RPS6KA2, SFRP2, BTG3, ADAMTS1, JAK2, TGFB1I1, PMP22 |
| 13 | GOTERM_BP_DIRECT | GO:0001501~skeletal system development | 1.86E-04 | BMP1, HEXB, FBN1, BCAN, SOX4, SOX9, COL5A2, TNFRSF11B, RPS6KA3, COL1A2, HOXA10, COL12A1, VCAN, COL1A1, PAPSS2, CDH11 |
| 14 | GOTERM_BP_DIRECT | GO:0030335~positive regulation of cell migration | 2.24E-04 | EGFR, LYN, AIF1, PODXL, FERMT3, HGF, MAPK1, PTP4A1, FGFR1OP, PDGFRB, HAS2, PDGFC, JAK2, COL1A1, SERPINB3, SEMA4D, THBS1, GPNMB, CYR61 |
| 15 | GOTERM_BP_DIRECT | GO:0043434~response to peptide hormone | 2.28E-04 | HHEX, CD55, BRAF, GNAI1, CTGF, GJA1, COL1A1, FAS, IRS1, NEFL |
| 16 | GOTERM_BP_DIRECT | GO:0043066~negative regulation of apoptotic process | 3.06E-04 | WNT5A, AIF1, SNCA, ARNT2, NFKBIA, BNIP3, SOX9, GREM1, EPCAM, EDNRB, RPS3A, FAS, THBS1, CYR61, EGFR, SOX10, PRAME, BRAF, BCL2A1, TP53, PIM1, ANXA5, CAPN3, PLAUR, ATF5, AMIGO2, RPS6KA3, PLK2, CCND2, PSEN2, SERPINB2, PDGFRB, SEMA4D, SLC9A1 |
| 17 | GOTERM_BP_DIRECT | GO:0045944~positive regulation of transcription from RNA polymerase II promoter | 3.48E-04 | MEF2C, NAMPT, FOSL2, LMO2, ELF4, HEXB, PPARG, ARNT2, PRRX1, TLR4, CBFB, EPCAM, GATA2, APP, CDKN2A, CDKN2B, SERPINE1, IFNG, YAP1, CYR61, PID1, EGFR, SOX10, MYO6, CTBP2, TP53, DMRT1, CD40, INHBA, HHEX, ZFPM2, ZNF382, WNT5A, BEX1, CTCFL, SOX4, NFKBIA, MYBL1, GREM1, SOX9, TCF7L2, BCL11A, HOXA10, MAF, CEBPA, FZD8, ESRRB, CEBPG, SMAD2, HGF, WWTR1, ATF5, RPS6KA3, SFRP2, CDON, HOXB9, IRF4, NFIB, SLC9A1 |
| 18 | GOTERM_BP_DIRECT | GO:0048469~cell maturation | 3.62E-04 | TUSC2, CEBPA, SOX10, GATA2, PLP1, PPARG, CBFB, BTK |
| 19 | GOTERM_BP_DIRECT | GO:0002576~platelet degranulation | 4.24E-04 | LGALS3BP, APP, RARRES2, CD36, LYN, CLU, SERPINE1, ABCC4, HGF, ITGB3, THBS1, PROS1, SRGN |
| 20 | GOTERM_BP_DIRECT | GO:0001568~blood vessel development | 4.30E-04 | MEF2C, ALDH1A2, CRKL, TGFBR2, COL1A2, COL1A1, TCF7L2, COL5A1 |
| 21 | GOTERM_BP_DIRECT | GO:0001934~positive regulation of protein phosphorylation | 4.53E-04 | EGFR, RARRES2, AIF1, CD40, ITGB3, SOX9, ANXA2, PLAUR, EDNRB, CCND2, SEMA4D, ABL1, GPNMB, LRRK2, CYR61 |
| 22 | GOTERM_BP_DIRECT | GO:0042060~wound healing | 5.32E-04 | WNT5A, EGFR, ERBB3, TGFBR2, SERPINB2, PDGFRB, DSP, LOX, CELSR1, ITGB3, SDC2, C6ORF89 |
| 23 | GOTERM_BP_DIRECT | GO:0048008~platelet-derived growth factor receptor signaling pathway | 5.79E-04 | PLAT, SGPL1, BCR, TIPARP, PDGFRB, PDGFC, JAK2 |
| 24 | GOTERM_BP_DIRECT | GO:0008219~cell death | 5.95E-04 | PTGER3, FOSL2, ZAK, CLU, BNIP3, FAF1, PMP22, EMP1 |
| 25 | GOTERM_BP_DIRECT | GO:0051017~actin filament bundle assembly | 7.01E-04 | AIF1, MYO1B, NEDD9, DPYSL3, PAWR, PLS3, LCP1 |
| 26 | GOTERM_BP_DIRECT | GO:0001558~regulation of cell growth | 7.22E-04 | NOV, HTRA1, CTGF, IGFBP7, IGFBP6, CAMK2D, HTRA3, CISH, IL17RB, NET1, CYR61 |
| 27 | GOTERM_BP_DIRECT | GO:0046718~viral entry into host cell | 7.22E-04 | ICAM1, LAMP1, CD55, VAMP8, CLDN1, SCARB2, SERPINB3, ITGB3, CXADR, MOG, DPP4 |
| 28 | GOTERM_BP_DIRECT | GO:0030208~dermatan sulfate biosynthetic process | 7.28E-04 | UST, DSEL, CSPG4, BCAN, VCAN |
| 29 | GOTERM_BP_DIRECT | GO:0030324~lung development | 7.97E-04 | WNT5A, CEBPA, EGFR, NPHP3, ALDH1A2, CTGF, CHI3L1, SMAD2, NOS3, ZFPM2, LOX |
| 30 | GOTERM_BP_DIRECT | GO:0042730~fibrinolysis | 8.53E-04 | PLAT, SERPINE1, SERPINB2, PROS1, ANXA2, PLAUR |
| 31 | GOTERM_BP_DIRECT | GO:0042542~response to hydrogen peroxide | 0.0010032 | GPX1, SLC8A1, STAR, PDGFRB, HBA2, COL1A1, HBA1, HBB, GLRX2 |
| 32 | GOTERM_BP_DIRECT | GO:1900026~positive regulation of substrate adhesion-dependent cell spreading | 0.0010047 | CRKL, BRAF, S100A10, HAS2, CALR, DOCK5, NET1 |
| 33 | GOTERM_BP_DIRECT | GO:0045429~positive regulation of nitric oxide biosynthetic process | 0.0012350 | EGFR, ICAM1, AIF1, CLU, IFNG, TLR4, JAK2, HBB |
| 34 | GOTERM_BP_DIRECT | GO:0045766~positive regulation of angiogenesis | 0.0012351 | WNT5A, TGFBR2, CHI3L1, HGF, GREM1, GATA2, SFRP2, CX3CR1, SERPINE1, RRAS, NOS3, THBS1, TERT |
| 35 | GOTERM_BP_DIRECT | GO:0000186~activation of MAPKK activity | 0.0018209 | EGFR, MAP3K4, CRKL, BRAF, ZAK, JAK2, LRRK2, MAP3K13 |
| 36 | GOTERM_BP_DIRECT | GO:0022408~negative regulation of cell-cell adhesion | 0.0018382 | SPINT2, PODXL, CDH1, JAK2, ABL1 |
| 37 | GOTERM_BP_DIRECT | GO:0006112~energy reserve metabolic process | 0.0018382 | PID1, KL, LEPR, GFPT2, MRAP2 |
| 38 | GOTERM_BP_DIRECT | GO:0015671~oxygen transport | 0.0018382 | HBA2, HBA1, HBG2, HBE1, HBB |
| 39 | GOTERM_BP_DIRECT | GO:0007417~central nervous system development | 0.0020450 | PAM, RPS6KA3, S100B, LYN, ARNT2, BCAN, RELN, PDGFC, VCAN, CELSR1, TIMP2, MOG, STK3 |
| 40 | GOTERM_BP_DIRECT | GO:0007050~cell cycle arrest | 0.0027153 | GAS2L3, ZAK, PRKAB1, TP53, GAS1, CALR, TCF7L2, INHBA, CDKN2A, CDKN2B, IFNG, ERN1, THBS1, ABL1 |
| 41 | GOTERM_BP_DIRECT | GO:0045930~negative regulation of mitotic cell cycle | 0.0028177 | EGFR, PTPN3, BTG3, GAS1, TIMP2, ABL1 |
| 42 | GOTERM_BP_DIRECT | GO:0071222~cellular response to lipopolysaccharide | 0.0028560 | MEF2C, WNT5A, ICAM1, PTGER3, STAR, TLR4, CD40, EDNRB, CD36, CX3CR1, IFNG, SERPINE1, ABL1 |
| 43 | GOTERM_BP_DIRECT | GO:0030336~negative regulation of cell migration | 0.0029210 | ARHGAP4, TMEFF2, PTPRK, IFITM1, ROBO1, SFRP2, SERPINE1, CX3CR1, RRAS, DPYSL3, THY1 |
| 44 | GOTERM_BP_DIRECT | GO:0002480~antigen processing and presentation of exogenous peptide antigen via MHC class I, TAP-independent | 0.0035972 | HLA-A, HLA-C, HLA-B, HLA-F |
| 45 | GOTERM_BP_DIRECT | GO:0071560~cellular response to transforming growth factor beta stimulus | 0.0036189 | WNT5A, MEF2C, STAR, FBN1, CX3CR1, COL1A1, ABL1, SOX9 |
| 46 | GOTERM_BP_DIRECT | GO:0035335~peptidyl-tyrosine dephosphorylation | 0.0036561 | DUSP4, PTPRK, PTPN3, PTP4A3, PTPRF, PTPRG, PTP4A1, PTPRN2, PTPN14, DNAJC6, PTPN21 |
| 47 | GOTERM_BP_DIRECT | GO:0090002~establishment of protein localization to plasma membrane | 0.0037614 | PKP3, MPP5, EFR3A, S100A10, CDH1, RAB13, CDH2 |
| 48 | GOTERM_BP_DIRECT | GO:0007420~brain development | 0.0039212 | CAST, BCR, STAR, CADM1, PTPRG, ARNT2, TGFBR2, WRN, ARL6, PCDH18, ZIC2, EML1, RELN, MARCKS, STMN1, FAS, NT5E |
| 49 | GOTERM_BP_DIRECT | GO:0002486~antigen processing and presentation of endogenous peptide antigen via MHC class I via ER pathway, TAP-independent | 0.0040110 | HLA-A, HLA-C, HLA-B |
| 50 | GOTERM_BP_DIRECT | GO:0071306~cellular response to vitamin E | 0.0040110 | PPARG, COL1A1, PAWR |
| 51 | GOTERM_BP_DIRECT | GO:0042127~regulation of cell proliferation | 0.0041711 | RBFOX2, ITK, TRNP1, BRAF, ERBB3, TGFBR2, NFKBIA, CD40, SOX9, BTK, HHEX, TNFRSF11B, BCL6, JAK2, FAS, ABL1 |
| 52 | GOTERM_BP_DIRECT | GO:0006915~apoptotic process | 0.0043280 | MEF2C, CADM1, SNCA, NFKBIA, BNIP3, GJA1, PAWR, GREM1, GLRX2, PEG10, CASP4, CDKN2A, CXCR4, IFNG, TNFRSF19, FAS, PRAME, GZMA, TGFBR2, RMDN3, TP53, CHI3L1, PIM1, NLRP2, CAPN3, ELMO2, STK3, MAPK1, RASSF5, RPS6KA3, TNFSF10, SFRP2, SH3KBP1, JAK2, FAF1 |
| 53 | GOTERM_BP_DIRECT | GO:0001570~vasculogenesis | 0.0044477 | HHEX, SGPL1, TGFBR2, TIPARP, HAS2, ZFPM2, YAP1, GJC1 |
| 54 | GOTERM_BP_DIRECT | GO:0050731~positive regulation of peptidyl-tyrosine phosphorylation | 0.0045109 | ICAM1, CD36, TP53, CSPG4, RELN, JAK2, SEMA4D, HGF, ITGB3, ABL1 |
| 55 | GOTERM_BP_DIRECT | GO:0071300~cellular response to retinoic acid | 0.0046316 | WNT5A, MEF2C, ALDH1A2, SERPINF1, LYN, PPARG, COL1A1, YAP1, SOX9 |
| 56 | GOTERM_BP_DIRECT | GO:0060324~face development | 0.0046404 | WNT5A, MAPK1, BBS4, ALDH1A2, BRAF |
| 57 | GOTERM_BP_DIRECT | GO:0097190~apoptotic signaling pathway | 0.0050458 | SGPL1, IFI27, TNFRSF11B, MLLT11, TLR4, PAWR, FAS, CD40, BTK |
| 58 | GOTERM_BP_DIRECT | GO:0051291~protein heterooligomerization | 0.0050458 | GLRB, BRAF, PRKAB1, CLDN1, HBA2, HBA1, CDH2, HBE1, HBB |
| 59 | GOTERM_BP_DIRECT | GO:0043406~positive regulation of MAP kinase activity | 0.0054123 | MEF2C, MAGED1, EGFR, PDGFRB, TPD52L1, PDGFC, CD40, LRRK2 |
| 60 | GOTERM_BP_DIRECT | GO:0010629~negative regulation of gene expression | 0.0055666 | MEF2C, BBS4, AIF1, TIPARP, GJA1, SMAD2, SERPINF1, ITGB8, CTGF, SFRP2, NME1, IFNG, TERT |
| 61 | GOTERM_BP_DIRECT | GO:0030318~melanocyte differentiation | 0.0056329 | MEF2C, MYO5A, EDNRB, SOX10, RAB27A |
| 62 | GOTERM_BP_DIRECT | GO:0030514~negative regulation of BMP signaling pathway | 0.0060158 | WNT5A, NBL1, HTRA1, SFRP2, HTRA3, GREM1, ABL1 |
| 63 | GOTERM_BP_DIRECT | GO:0002474~antigen processing and presentation of peptide antigen via MHC class I | 0.0060370 | HLA-DQB1, HLA-A, HLA-C, HLA-B, CALR, HLA-F |
| 64 | GOTERM_BP_DIRECT | GO:2000379~positive regulation of reactive oxygen species metabolic process | 0.0060370 | PID1, CD36, TGFBR2, TP53, PDGFRB, THBS1 |
| 65 | GOTERM_BP_DIRECT | GO:0032092~positive regulation of protein binding | 0.0065256 | WNT5A, CTHRC1, PLK2, EPB41, LRRK2, TCF7L2, STK3, TERT |
| 66 | GOTERM_BP_DIRECT | GO:0033993~response to lipid | 0.0066851 | GATA2, CD36, PPARG, MOG |
| 67 | GOTERM_BP_DIRECT | GO:0050885~neuromuscular process controlling balance | 0.0067066 | RBFOX2, APP, SLC1A3, BCR, HEXB, ABL1, NEFL |
| 68 | GOTERM_BP_DIRECT | GO:0022617~extracellular matrix disassembly | 0.0069921 | BMP1, HTRA1, FBN1, BCAN, CDH1, CAPN2, TIMP2, ADAMTS5, LCP1 |
| 69 | GOTERM_BP_DIRECT | GO:0060021~palate development | 0.0075569 | WNT5A, MEF2C, FRAS1, INHBA, SGPL1, TGFBR2, TIPARP, PRRX1, SMAD2 |
| 70 | GOTERM_BP_DIRECT | GO:0090090~negative regulation of canonical Wnt signaling pathway | 0.0077154 | WNT5A, CTHRC1, SOX10, PPP2R3A, IGFBP6, CDH2, GREM1, SOX9, WWTR1, TCF7L2, STK3, FZD6, NPHP3, SFRP2 |
| 71 | GOTERM_BP_DIRECT | GO:0030512~negative regulation of transforming growth factor beta receptor signaling pathway | 0.0078009 | CAV2, PEG10, HTRA1, TGFBR2, SMAD2, TGFB1I1, HTRA3, PMEPA1 |
| 72 | GOTERM_BP_DIRECT | GO:0008626~granzyme-mediated apoptotic signaling pathway | 0.0078251 | LAMP1, BNIP3, SRGN |
| 73 | GOTERM_BP_DIRECT | GO:0044691~tooth eruption | 0.0078251 | ADAMTS1, COL1A1, ADAMTS5 |
| 74 | GOTERM_BP_DIRECT | GO:0010718~positive regulation of epithelial to mesenchymal transition | 0.0078512 | TGFBR2, SMAD2, TGFB1I1, COL1A1, SERPINB3, WWTR1 |
| 75 | GOTERM_BP_DIRECT | GO:0009636~response to toxic substance | 0.0080510 | MAPK1, NUPR1, LYN, EPHX2, PDGFRB, EPHX1, CDH1, FAS, NEFL, RAD51 |
| 76 | GOTERM_BP_DIRECT | GO:0000902~cell morphogenesis | 0.0085034 | EGFR, CAP2, CLU, DMRT1, BCL6, YAP1, HGF, GREM1 |
| 77 | GOTERM_BP_DIRECT | GO:0045669~positive regulation of osteoblast differentiation | 0.0085034 | MEF2C, CEBPA, CTHRC1, IFITM1, SFRP2, GJA1, HGF, CYR61 |
| 78 | GOTERM_BP_DIRECT | GO:0071333~cellular response to glucose stimulus | 0.0092512 | MEF2C, ICAM1, SERPINF1, STAR, NME1, ERN1, SOX4, FAS |
| 79 | GOTERM_BP_DIRECT | GO:0060337~type I interferon signaling pathway | 0.0092512 | IFI27, IFITM1, HLA-A, HLA-C, OAS1, HLA-B, IRF4, HLA-F |
| 80 | GOTERM_BP_DIRECT | GO:0071345~cellular response to cytokine stimulus | 0.0094304 | PID1, CXCR4, NFKBIA, DPYSL3, ITGA4 |
| 81 | GOTERM_BP_DIRECT | GO:0009952~anterior/posterior pattern specification | 0.0094579 | ALDH1A2, HHEX, HOXB2, CRKL, CDON, HOXA10, SMAD2, HOXB9, CELSR1 |
| 82 | GOTERM_BP_DIRECT | GO:0014068~positive regulation of phosphatidylinositol 3-kinase signaling | 0.0100461 | ERBB3, PDGFRB, RELN, PDGFC, JAK2, SEMA4D, HGF, SOX9 |
| 83 | GOTERM_BP_DIRECT | GO:0007507~heart development | 0.0108623 | NRP2, MEF2C, PAM, ERBB3, FBN1, PPARG, TGFBR2, GJA1, SOX4, CXADR, GATA2, CRKL, ROBO1, RPS6KA2, LOX |
| 84 | GOTERM_BP_DIRECT | GO:0007157~heterophilic cell-cell adhesion via plasma membrane cell adhesion molecules | 0.0110438 | ALCAM, ICAM1, AMIGO2, CADM1, CDH2, CXADR, CD200 |
| 85 | GOTERM_BP_DIRECT | GO:0034332~adherens junction organization | 0.0112331 | CADM1, DSP, CDH1, CDH2, CDH11, CDH6 |
| 86 | GOTERM_BP_DIRECT | GO:0045892~negative regulation of transcription, DNA-templated | 0.0116860 | WNT5A, PPARG, NOSTRIN, SOX9, CALR, GREM1, TCF7L2, ZIC2, MAGED1, CDKN2A, ATP8B1, BCL6, LOXL2, CEBPA, RBFOX2, SOX10, PTPRK, ZNF280B, CTBP2, PRAME, TP53, ZHX2, TLE1, SMAD2, BASP1, CAPN3, ATF5, HHEX, SFRP2, ZFPM2 |
| 87 | GOTERM_BP_DIRECT | GO:0098869~cellular oxidant detoxification | 0.0117833 | GPX1, NXN, GSTT1, HBA2, GPX8, HBA1, HBB, MGST2 |
| 88 | GOTERM_BP_DIRECT | GO:0001942~hair follicle development | 0.0125508 | EGFR, INHBA, TNFRSF19, CELSR1, SOX9, FZD6 |
| 89 | GOTERM_BP_DIRECT | GO:0030206~chondroitin sulfate biosynthetic process | 0.0127095 | CHST7, CSPG4, BCAN, VCAN, CHST15 |
| 90 | GOTERM_BP_DIRECT | GO:0071279~cellular response to cobalt ion | 0.0127226 | SERPINF1, BNIP3, FAS |
| 91 | GOTERM_BP_DIRECT | GO:0090027~negative regulation of monocyte chemotaxis | 0.0127226 | NOV, NBL1, GREM1 |
| 92 | GOTERM_BP_DIRECT | GO:0048661~positive regulation of smooth muscle cell proliferation | 0.0127288 | EGFR, NAMPT, AIF1, TGFBR2, CAMK2D, PDGFRB, ABCC4, THBS1 |
| 93 | GOTERM_BP_DIRECT | GO:0071260~cellular response to mechanical stimulus | 0.0133864 | EGFR, GJA1, BNIP3, TLR4, COL1A1, FAS, CD40, SOX9, SLC9A1 |
| 94 | GOTERM_BP_DIRECT | GO:0030194~positive regulation of blood coagulation | 0.0135772 | CD36, ENPP4, SERPINE1, THBS1 |
| 95 | GOTERM_BP_DIRECT | GO:0030207~chondroitin sulfate catabolic process | 0.0135772 | HEXB, CSPG4, BCAN, VCAN |
| 96 | GOTERM_BP_DIRECT | GO:0042981~regulation of apoptotic process | 0.0139013 | BMP1, TP53, GAS1, WRN, CALR, SOX9, MAGED1, TNFRSF11B, RASSF5, CASP4, INHBE, NME1, BCL6, JAK2, FAS, GDF15 |
| 97 | GOTERM_BP_DIRECT | GO:0045907~positive regulation of vasoconstriction | 0.0139692 | EGFR, ICAM1, HRH1, PTGER3, GJA1, ABL1 |
| 98 | GOTERM_BP_DIRECT | GO:0060291~long-term synaptic potentiation | 0.0139692 | MAPK1, BRAF, PLK2, S100B, SNCA, RELN |
| 99 | GOTERM_BP_DIRECT | GO:0001666~response to hypoxia | 0.0139930 | PLAT, PAM, ARNT2, TGFBR2, BNIP3, PDLIM1, CAPN2, SDC2, PLOD2, CXCR4, PSEN2, CAMK2D, THBS1, LOXL2, DPP4 |
| 100 | GOTERM_BP_DIRECT | GO:0005975~carbohydrate metabolic process | 0.0141048 | MGAT4B, GALNT3, LDHB, MGAT4A, KL, HEXB, CHI3L1, DHDH, MAN2A2, GALM, CHST6, GFPT2, PGM1, AKR7A2 |
| 101 | GOTERM_BP_DIRECT | GO:0030154~cell differentiation | 0.0142427 | ZAK, CADM1, ELF4, BEX1, GPM6B, SLFN5, DLK1, MYBL1, NHS, GLRX2, ZIC2, CCHCR1, PEG10, CTGF, RPS3A, SH2B3, PRAME, RMDN3, TGFBR2, TP53, GTSF1, HHEX, INHBA, UHRF2, NXN, JAK2, TGFB1I1, AGR3 |
| 102 | GOTERM_BP_DIRECT | GO:0006897~endocytosis | 0.0143778 | STON2, LY75, RAB7A, APP, STON1, MYO6, NME1, RIN2, SH3KBP1, NOSTRIN, EHD1, LRRK2 |
| 103 | GOTERM_BP_DIRECT | GO:0050776~regulation of immune response | 0.0153679 | ICAM1, IFITM1, HLA-A, COL1A2, BCL6, HLA-C, COL1A1, HLA-B, ITGA4, CD40, CXADR, CD200, HLA-F |
| 104 | GOTERM_BP_DIRECT | GO:0033627~cell adhesion mediated by integrin | 0.0165120 | NOV, ICAM1, FBN1, ITGB3 |
| 105 | GOTERM_BP_DIRECT | GO:0007010~cytoskeleton organization | 0.0168087 | ARHGAP4, ABLIM1, MAST4, CAP2, ZAK, SVIL, SH3KBP1, NEDD9, TUBB6, SOX9, PALLD, ELMO2, THY1 |
| 106 | GOTERM_BP_DIRECT | GO:0048468~cell development | 0.01712085 | MAF, INHBA, BMP1, INHBE, GDF15, GJC1 |
| 107 | GOTERM_BP_DIRECT | GO:0060548~negative regulation of cell death | 0.0184936 | NOV, CTGF, SOX4, BNIP3, ZFPM2, AGR3, CYR61 |
| 108 | GOTERM_BP_DIRECT | GO:0048146~positive regulation of fibroblast proliferation | 0.0184936 | WNT5A, EGFR, FOSL2, PDGFRB, PDGFC, ABL1, ANXA2 |
| 109 | GOTERM_BP_DIRECT | GO:0043388~positive regulation of DNA binding | 0.0188556 | NME1, CEBPG, JAK2, IRF4, PLAUR |
| 110 | GOTERM_BP_DIRECT | GO:2000352~negative regulation of endothelial cell apoptotic process | 0.0188556 | ICAM1, BRAF, SERPINE1, ABL1, TERT |
| 111 | GOTERM_BP_DIRECT | GO:0090200~positive regulation of release of cytochrome c from mitochondria | 0.0188556 | TNFSF10, MLLT11, TP53, BNIP3, PLAUR |
| 112 | GOTERM_BP_DIRECT | GO:0097191~extrinsic apoptotic signaling pathway | 0.0188596 | INHBA, MLLT11, IFNG, G0S2, JAK2, FAS |
| 113 | GOTERM_BP_DIRECT | GO:0006024~glycosaminoglycan biosynthetic process | 0.0188596 | GALNT5, SLC35D2, PDGFRB, SDC2, HS3ST3B1, HS2ST1 |
| 114 | GOTERM_BP_DIRECT | GO:0045444~fat cell differentiation | 0.0195748 | CEBPA, PID1, ATF5, BBS4, GPX1, BBS9, ARL6, TCF7L2 |
| 115 | GOTERM_BP_DIRECT | GO:0009749~response to glucose | 0.0195748 | SLC8A1, CTGF, TGFBR2, COL6A2, GJA1, SMAD2, THBS1, TCF7L2 |
| 116 | GOTERM_BP_DIRECT | GO:0060216~definitive hemopoiesis | 0.0197733 | GATA2, LYL1, MFAP5, CBFB |
| 117 | GOTERM_BP_DIRECT | GO:0030889~negative regulation of B cell proliferation | 0.0197733 | CDKN2A, LYN, MNDA, PAWR |
| 118 | GOTERM_BP_DIRECT | GO:0008631~intrinsic apoptotic signaling pathway in response to oxidative stress | 0.0197733 | GPX1, JAK2, ARL6IP5, PRODH |
| 119 | GOTERM_BP_DIRECT | GO:0061036~positive regulation of cartilage development | 0.0197733 | WNT5A, BMP1, SOX9, CYR61 |
| 120 | GOTERM_BP_DIRECT | GO:0007568~aging | 0.0199405 | CAST, EDNRB, SERPINF1, KL, CTGF, TGFBR2, CLU, BCL2A1, SNCA, FAS, WRN, TIMP2, LOXL2, MOG, GLRX2 |
| 121 | GOTERM_BP_DIRECT | GO:0007005~mitochondrion organization | 0.0223383 | CEBPA, CAV2, CHCHD10, NOS3, RAB38, LRRK2, CXADR, TERT |
| 122 | GOTERM_BP_DIRECT | GO:0007264~small GTPase mediated signal transduction | 0.0229974 | RAB7A, MRAS, IQGAP2, ARL6, DOCK5, ARHGAP30, RND3, RAB34, RIN2, RAB24, RRAS, RAB13, RAB38, LRRK2, RHOBTB3, RAB27A, ARL4A |
| 123 | GOTERM_BP_DIRECT | GO:0035567~non-canonical Wnt signaling pathway | 0.0233629 | WNT5A, FZD8, SFRP2, FZD6 |
| 124 | GOTERM_BP_DIRECT | GO:0071285~cellular response to lithium ion | 0.0233629 | CEBPA, CDH1, FAS, CALR |
| 125 | GOTERM_BP_DIRECT | GO:0060907~positive regulation of macrophage cytokine production | 0.0254320 | WNT5A, CD36, TLR4 |
| 126 | GOTERM_BP_DIRECT | GO:0014909~smooth muscle cell migration | 0.0254320 | PLAT, NOV, ITGB3 |
| 127 | GOTERM_BP_DIRECT | GO:0050766~positive regulation of phagocytosis | 0.0265700 | GATA2, BCR, CALR, PROS1, RAB27A |
| 128 | GOTERM_BP_DIRECT | GO:0010942~positive regulation of cell death | 0.0265700 | HBA2, FAF1, HBA1, HBB, PRODH |
| 129 | GOTERM_BP_DIRECT | GO:0045732~positive regulation of protein catabolic process | 0.0268731 | WNT5A, RAB7A, PPP2R3A, PLK2, TIPARP, GJA1, SOX9 |
| 130 | GOTERM_BP_DIRECT | GO:0019882~antigen processing and presentation | 0.0269604 | RAB34, IFNG, HLA-A, HLA-C, HLA-B, RAB27A |
| 131 | GOTERM_BP_DIRECT | GO:0061512~protein localization to cilium | 0.0272809 | BBS4, BBS9, ARL6, EHD1 |
| 132 | GOTERM_BP_DIRECT | GO:0031663~lipopolysaccharide-mediated signaling pathway | 0.0295033 | MAPK1, LYN, NFKBIA, TLR4, NOS3 |
| 133 | GOTERM_BP_DIRECT | GO:0001503~ossification | 0.0303998 | EGFR, BMP1, IFITM1, CTGF, GPM6B, SOX9, COL5A2, CDH11 |
| 134 | GOTERM_BP_DIRECT | GO:0045666~positive regulation of neuron differentiation | 0.0303998 | MEF2C, NBL1, GATA2, CDON, IFNG, HMG20B, BCL6, TIMP2 |
| 135 | GOTERM_BP_DIRECT | GO:0030574~collagen catabolic process | 0.0308298 | PEPD, COL1A2, COL6A2, COL12A1, COL1A1, COL5A2, COL5A1 |
| 136 | GOTERM_BP_DIRECT | GO:0007049~cell cycle | 0.0310078 | TUSC2, DBF4B, RABGAP1, TRNP1, GNAI1, TP53, PIM1, CTCFL, HMG20B, TRIM21, MAPK1, RPS6KA3, UHRF2, CCND2, PTP4A1 |
| 137 | GOTERM_BP_DIRECT | GO:0031668~cellular response to extracellular stimulus | 0.0315260 | CDKN2B, LYN, AIF1, SFRP2 |
| 138 | GOTERM_BP_DIRECT | GO:0035556~intracellular signal transduction | 0.0316297 | ITK, BCR, ZAK, DMRT1, CSPG4, SMAD2, ARHGEF12, STK3, CISH, BTK, PRKAR2B, MAST4, RASSF5, RPS6KA3, MAP3K4, CRKL, CTGF, RPS6KA2, PSEN2, SH2B3, JAK2, STMN1, LRRK2, NET1 |
| 139 | GOTERM_BP_DIRECT | GO:0035019~somatic stem cell population maintenance | 0.0329434 | GATA2, BRAF, ZHX2, SOX4, SMAD2, YAP1, SOX9 |
| 140 | GOTERM_BP_DIRECT | GO:0046825~regulation of protein export from nucleus | 0.0330875 | IFI27, CDKN2A, PTPN14 |
| 141 | GOTERM_BP_DIRECT | GO:0010737~protein kinase A signaling | 0.0330875 | FBN1, RAB13, LCP1 |
| 142 | GOTERM_BP_DIRECT | GO:2000020~positive regulation of male gonad development | 0.0330875 | DMRT1, ZFPM2, SOX9 |
| 143 | GOTERM_BP_DIRECT | GO:0060993~kidney morphogenesis | 0.0330875 | NPHP3, SOX4, WWTR1 |
| 144 | GOTERM_BP_DIRECT | GO:0016337~single organismal cell-cell adhesion | 0.0338545 | EGFR, ICAM1, ROPN1B, KIRREL, PKP3, DSP, CDH1, SOX9, THY1 |
| 145 | GOTERM_BP_DIRECT | GO:0048812~neuron projection morphogenesis | 0.0342961 | EGFR, NBL1, CLU, GJA1, LRRK2, NEFL |
| 146 | GOTERM_BP_DIRECT | GO:0000122~negative regulation of transcription from RNA polymerase II promoter | 0.0346596 | MEF2C, SNCA, PPARG, PRRX1, PAWR, SOX9, CALR, TCEAL1, TCF7L2, GATA2, EDNRB, BCL11A, IFNG, NR2F6, BCL6, CEBPA, MAF, FZD8, ZNF280B, CTBP2, HIST1H1C, TP53, ZHX2, DMRT1, LMCD1, TLE1, SMAD2, WWTR1, RBBP8, HHEX, IFI27, PHF19, CD36, TGIF1, ZFPM2, SEMA4D, ZNF382, NFIB |
| 147 | GOTERM_BP_DIRECT | GO:0002088~lens development in camera-type eye | 0.0359232 | WNT5A, CDON, TGFBR2, GJA1, NHS |
| 148 | GOTERM_BP_DIRECT | GO:0010951~negative regulation of endopeptidase activity | 0.0359316 | CAST, APP, SERPINF1, LXN, SPINT2, SERPINE1, SERPINB2, SERPINB3, TIMP2, PROS1 |
| 149 | GOTERM_BP_DIRECT | GO:0006919~activation of cysteine-type endopeptidase activity involved in apoptotic process | 0.0360722 | IFI27, TNFSF10, CDKN2A, ROBO1, PPARG, SNCA, JAK2, FAS |
| 150 | GOTERM_BP_DIRECT | GO:0001892~embryonic placenta development | 0.0360954 | CEBPA, EGFR, GATA2, ESRRB |
| 151 | GOTERM_BP_DIRECT | GO:0007520~myoblast fusion | 0.0360954 | CAST, CDON, TANC1, CAPN2 |
| 152 | GOTERM_BP_DIRECT | GO:0042744~hydrogen peroxide catabolic process | 0.0360954 | GPX1, HBA2, HBA1, HBB |
| 153 | GOTERM_BP_DIRECT | GO:0006491~N-glycan processing | 0.0360954 | MAN2A2, MGAT4B, MGAT4A, MAN1A1 |
| 154 | GOTERM_BP_DIRECT | GO:0007275~multicellular organism development | 0.0364639 | WNT5A, LMO2, CADM1, MRAS, DZIP1, LEPR, TDRG1, HOOK1, CCHCR1, CREG1, HOXA10, SPRED1, STRBP, NFE2, BMP1, TP53, PIM1, TLE1, CD40, FZD6, HOXB2, SERPINF1, SFRP2, PTP4A1, TGIF1, RPS4Y1, HIVEP2, VCAN, EMP1 |
| 155 | GOTERM_BP_DIRECT | GO:0007165~signal transduction | 0.0367208 | NAMPT, PPARG, IGFBP6, GJA1, IQGAP2, FGF13, ARHGAP4, TNFRSF11B, NR2F6, FAS, NET1, EGFR, PTPRK, BCR, LYN, MPP1, PRKAB1, PI4KA, TLE1, IRS1, STK3, PLAUR, ARHGAP30, MAPK1, GNB2, RIN2, GNB5, PDGFRB, INPP4B, STMN1, ERBB3, NEDD9, NOSTRIN, SOX9, GREM1, APBB1IP, PDAP1, ALCAM, LGALS3BP, BCL11A, SH2B3, OR51B4, NMU, LY75, ITK, CAP2, ANXA5, CAPN3, GMFB, MYO10, RPS6KA3, TNFSF10, RPS6KA2, HIVEP2, JAK2, GDF15 |
| 156 | GOTERM_BP_DIRECT | GO:0070374~positive regulation of ERK1 and ERK2 cascade | 0.0386635 | EGFR, ICAM1, CD36, BRAF, CTGF, CHI3L1, CAMK2D, PDGFRB, TLR4, PDGFC, PRKCDBP, ABL1, GPNMB |
| 157 | GOTERM_BP_DIRECT | GO:0050900~leukocyte migration | 0.0392736 | ICAM1, LYN, PODXL, COL1A2, L1CAM, COL1A1, ITGA4, ITGB3, CXADR, PROS1 |
| 158 | GOTERM_BP_DIRECT | GO:0072593~reactive oxygen species metabolic process | 0.0394118 | CTGF, EPHX2, BNIP3, LRRK2, CYR61 |
| 159 | GOTERM_BP_DIRECT | GO:0071230~cellular response to amino acid stimulus | 0.0398103 | EGFR, COL1A2, PDGFC, COL1A1, CAPN2, COL5A2 |
| 160 | GOTERM_BP_DIRECT | GO:0007399~nervous system development | 0.0408398 | MEF2C, RBFOX2, GLRB, NINJ2, BEX1, GPM6B, FGF13, L1CAM, PCDHA1, SIGMAR1, PCDH18, GMFB, INHBA, NBL1, EDNRB, APP, ROBO1, GFRA1 |
| 161 | GOTERM_BP_DIRECT | GO:0032402~melanosome transport | 0.04098510 | MYO5A, BBS4, ARL6, RAB27A |
| 162 | GOTERM_BP_DIRECT | GO:0032355~response to estradiol | 0.0410212 | WNT5A, ALDH1A2, PAM, PTGER3, CTGF, ARNT2, PDGFRB, COL1A1, CALR, RBBP8 |
| 163 | GOTERM_BP_DIRECT | GO:0021795~cerebral cortex cell migration | 0.0415133 | EGFR, CX3CR1, FGF13 |
| 164 | GOTERM_BP_DIRECT | GO:0070886~positive regulation of calcineurin-NFAT signaling cascade | 0.0415133 | ERBB3, LMCD1, SLC9A1 |
| 165 | GOTERM_BP_DIRECT | GO:0000075~cell cycle checkpoint | 0.0415133 | ZAK, CCNG2, RBBP8 |
| 166 | GOTERM_BP_DIRECT | GO:0001764~neuron migration | 0.0431643 | MEF2C, MATN2, BBS4, GATA2, CXCR4, GJA1, FGF13, RELN, CELSR1 |
| 167 | GOTERM_BP_DIRECT | GO:0043154~negative regulation of cysteine-type endopeptidase activity involved in apoptotic process | 0.0449082 | GPX1, RPS6KA3, SFRP2, SNCA, HGF, THBS1, MAGEA3 |
| 168 | GOTERM_BP_DIRECT | GO:0071456~cellular response to hypoxia | 0.0452101 | ICAM1, SLC8A1, S100B, TP53, BNIP3, FAS, MPL, TERT, SLC9A1 |
| 169 | GOTERM_BP_DIRECT | GO:0055093~response to hyperoxia | 0.0461899 | WNT5A, PDGFRB, BNIP3, COL1A1 |
| 170 | GOTERM_BP_DIRECT | GO:0098609~cell-cell adhesion | 0.0468144 | CAST, MYO6, CNN3, MYO1B, CALD1, PI4KA, PDLIM1, TAGLN2, ELMO2, ANXA2, EPCAM, TMEM47, CRKL, STXBP6, TMOD3, EHD1, PHLDB2 |
| 171 | GOTERM_BP_DIRECT | GO:0033574~response to testosterone | 0.0469477 | WNT5A, NME1, MTAP, CALR, THBS1 |
| 172 | GOTERM_BP_DIRECT | GO:0045893~positive regulation of transcription, DNA-templated | 0.0470357 | MEF2C, WNT5A, ELF4, ARNT2, PPARG, SOX4, CTCFL, CDH1, TBP, MYBL1, SOX9, ZIC2, MAGED1, CDKN2A, LYL1, NFE2, TP53, SMAD2, CAPN3, ATF5, INHBA, MAPK1, MLLT11, ZNF711, CAND2, COL1A1, IRF4, TGFB1I1 |
| 173 | GOTERM_BP_DIRECT | GO:0000226~microtubule cytoskeleton organization | 0.0475867 | GAS2L3, BBS4, DYNC1LI2, EML1, CAMSAP2, NEFL, PHLDB2 |
| 174 | GOTERM_BP_DIRECT | GO:0050853~B cell receptor signaling pathway | 0.0490369 | MEF2C, MAPK1, LYN, MNDA, ABL1, BTK |
| 1 | KEGG_PATHWAY | hsa05144:Malaria | 4.86E-06 | GYPB, ICAM1, CD36, IFNG, HBA2, TLR4, HBA1, HGF, CD40, THBS1, HBB, SDC2 |
| 2 | KEGG_PATHWAY | hsa04514:Cell adhesion molecules (CAMs) | 1.04E-04 | HLA-DQB1, ICAM1, PTPRF, CADM1, HLA-A, CDH1, HLA-C, L1CAM, HLA-B, CD40, ITGA4, CDH2, SDC2, HLA-F, ALCAM, ITGB8, CLDN1, VCAN |
| 3 | KEGG_PATHWAY | hsa05416:Viral myocarditis | 1.25E-04 | HLA-DQB1, ICAM1, CD55, HLA-A, SGCD, HLA-C, HLA-B, CD40, ABL1, CXADR, HLA-F |
| 4 | KEGG_PATHWAY | hsa05200:Pathways in cancer | 1.27E-04 | WNT5A, GNAI1, ARNT2, PPARG, NFKBIA, FGF13, CDH1, TCF7L2, EDNRB, CDKN2A, CDKN2B, CXCR4, FAS, EGFR, CEBPA, FZD8, PTGER3, BCR, CTBP2, BRAF, TGFBR2, TP53, SMAD2, HGF, ARHGEF12, FZD6, RAD51, MAPK1, RASSF5, CRKL, GNB2, GNB5, PDGFRB, ABL1 |
| 5 | KEGG_PATHWAY | hsa05166:HTLV-I infection | 2.19E-04 | WNT5A, HLA-DQB1, MRAS, NFKBIA, TBP, MYBL1, CALR, CDKN2A, CDKN2B, RRAS, TERT, ICAM1, FZD8, TGFBR2, HLA-A, TP53, SMAD2, HLA-C, CD40, HLA-B, VDAC2, FZD6, HLA-F, CCND2, PDGFRB |
| 6 | KEGG_PATHWAY | hsa05205:Proteoglycans in cancer | 3.20E-04 | WNT5A, EGFR, CAV2, FZD8, BRAF, ERBB3, MRAS, TP53, TLR4, HGF, ITGB3, ARHGEF12, SDC2, PLAUR, FZD6, MAPK1, CAMK2D, RRAS, FAS, THBS1, SLC9A1 |
| 7 | KEGG_PATHWAY | hsa04510:Focal adhesion | 4.69E-04 | EGFR, CAV2, BRAF, HGF, ITGA4, ITGB3, CAPN2, COL5A2, COL5A1, MAPK1, CRKL, ITGB8, CCND2, COL1A2, COL6A2, PDGFRB, RELN, PDGFC, COL1A1, THBS1, PARVA |
| 8 | KEGG_PATHWAY | hsa05330:Allograft rejection | 8.21E-04 | HLA-DQB1, IFNG, HLA-A, HLA-C, HLA-B, FAS, CD40, HLA-F |
| 9 | KEGG_PATHWAY | hsa05220:Chronic myeloid leukemia | 8.82E-04 | MAPK1, CDKN2A, CRKL, BCR, CTBP2, GAB2, BRAF, TGFBR2, TP53, NFKBIA, ABL1 |
| 10 | KEGG_PATHWAY | hsa04068:FoxO signaling pathway | 0.00165018 | EGFR, GABARAPL2, BRAF, TGFBR2, PRKAB1, BNIP3, SMAD2, CCNG2, IRS1, MAPK1, TNFSF10, PLK2, CDKN2B, CCND2, BCL6 |
| 11 | KEGG_PATHWAY | hsa04940:Type I diabetes mellitus | 0.00179240 | HLA-DQB1, PTPRN2, IFNG, HLA-A, HLA-C, HLA-B, FAS, HLA-F |
| 12 | KEGG_PATHWAY | hsa05332:Graft-versus-host disease | 0.00240251 | HLA-DQB1, IFNG, HLA-A, HLA-C, HLA-B, FAS, HLA-F |
| 13 | KEGG_PATHWAY | hsa05218:Melanoma | 0.00302245 | EGFR, MAPK1, CDKN2A, BRAF, TP53, PDGFRB, FGF13, CDH1, PDGFC, HGF |
| 14 | KEGG_PATHWAY | hsa04611:Platelet activation | 0.00352563 | LYN, GNAI1, FERMT3, ITGB3, ARHGEF12, COL5A2, APBB1IP, COL5A1, BTK, MAPK1, VAMP8, COL1A2, NOS3, COL1A1 |
| 15 | KEGG_PATHWAY | hsa04512:ECM-receptor interaction | 0.00374895 | CD36, ITGB8, COL1A2, COL6A2, RELN, COL1A1, ITGA4, ITGB3, THBS1, COL5A2, COL5A1 |
| 16 | KEGG_PATHWAY | hsa04722:Neurotrophin signaling pathway | 0.00497977 | BRAF, TP53, NFKBIA, IRS1, MAGED1, MAPK1, RPS6KA3, CRKL, RPS6KA2, PSEN2, CAMK2D, SH2B3, ABL1 |
| 17 | KEGG_PATHWAY | hsa05202:Transcriptional misregulation in cancer | 0.00527367 | MAF, PLAT, CEBPA, MEF2C, LMO2, ARNT2, PPARG, TGFBR2, BCL2A1, TP53, CD40, HHEX, NUPR1, LYL1, CCND2, BCL6 |
| 18 | KEGG_PATHWAY | hsa05216:Thyroid cancer | 0.00700013 | MAPK1, BRAF, PPARG, TP53, CDH1, TCF7L2 |
| 19 | KEGG_PATHWAY | hsa05219:Bladder cancer | 0.00733047 | EGFR, MAPK1, CDKN2A, BRAF, TP53, CDH1, THBS1 |
| 20 | KEGG_PATHWAY | hsa04350:TGF-beta signaling pathway | 0.00922756 | MAPK1, NBL1, INHBA, CDKN2B, INHBE, TGFBR2, IFNG, TGIF1, SMAD2, THBS1 |
| 21 | KEGG_PATHWAY | hsa04151:PI3K-Akt signaling pathway | 0.01200868 | EGFR, PPP2R3A, TP53, TLR4, FGF13, HGF, ITGA4, ITGB3, COL5A2, IRS1, COL5A1, MAPK1, GNB2, ITGB8, CCND2, COL1A2, COL6A2, GNB5, PDGFRB, RELN, PDGFC, NOS3, JAK2, COL1A1, THBS1 |
| 22 | KEGG_PATHWAY | hsa04390:Hippo signaling pathway | 0.01217073 | WNT5A, FZD8, TGFBR2, MPP5, CDH1, SMAD2, WWTR1, TCF7L2, STK3, FZD6, CTGF, CCND2, SERPINE1, YAP1 |
| 23 | KEGG_PATHWAY | hsa05143:African trypanosomiasis | 0.01217764 | ICAM1, IFNG, HBA2, FAS, HBA1, HBB |
| 24 | KEGG_PATHWAY | hsa04010:MAPK signaling pathway | 0.01240659 | MEF2C, EGFR, ZAK, BRAF, MRAS, TGFBR2, TP53, FGF13, STK3, DUSP4, MAPK1, RPS6KA3, MAP3K4, CRKL, RPS6KA2, RRAS, PDGFRB, STMN1, FAS, MAP3K13 |
| 25 | KEGG_PATHWAY | hsa05142:Chagas disease (American trypanosomiasis) | 0.01297331 | MAPK1, GNA15, GNAI1, TGFBR2, SERPINE1, IFNG, NFKBIA, SMAD2, TLR4, FAS, CALR |
| 26 | KEGG_PATHWAY | hsa04145:Phagosome | 0.01348575 | HLA-DQB1, RAB7A, DYNC1LI2, HLA-A, TLR4, HLA-C, ITGB3, HLA-B, CALR, HLA-F, LAMP1, CD36, TUBB6, THBS1 |
| 27 | KEGG_PATHWAY | hsa04015:Rap1 signaling pathway | 0.01722764 | EGFR, BRAF, GNAI1, MRAS, CDH1, FGF13, ITGB3, HGF, APBB1IP, MAPK1, RASSF5, PFN2, CRKL, RRAS, PDGFRB, PDGFC, THBS1 |
| 28 | KEGG_PATHWAY | hsa05212:Pancreatic cancer | 0.02012910 | EGFR, MAPK1, CDKN2A, BRAF, TGFBR2, TP53, SMAD2, RAD51 |
| 29 | KEGG_PATHWAY | hsa05320:Autoimmune thyroid disease | 0.02248832 | HLA-DQB1, HLA-A, HLA-C, HLA-B, FAS, CD40, HLA-F |
| 30 | KEGG_PATHWAY | hsa05412:Arrhythmogenic right ventricular cardiomyopathy (ARVC) | 0.03102463 | ITGB8, SGCD, GJA1, DSP, ITGA4, CDH2, ITGB3, TCF7L2 |
| 31 | KEGG_PATHWAY | hsa04810:Regulation of actin cytoskeleton | 0.03557244 | EGFR, BRAF, MRAS, IQGAP2, FGF13, ITGA4, ITGB3, ARHGEF12, MAPK1, PFN2, CRKL, ITGB8, RRAS, PDGFRB, PDGFC, SLC9A1 |
| 32 | KEGG_PATHWAY | hsa05146:Amoebiasis | 0.03669637 | RAB7A, GNA15, IFNG, COL1A2, SERPINB2, TLR4, COL1A1, SERPINB3, COL5A2, COL5A1 |
| 33 | KEGG_PATHWAY | hsa04612:Antigen processing and presentation | 0.04268462 | HLA-DQB1, LGMN, IFNG, HLA-A, HLA-C, HLA-B, CALR, HLA-F |
| 34 | KEGG_PATHWAY | hsa04144:Endocytosis | 0.04885575 | EGFR, RAB7A, CAV2, CHMP4C, ERBB3, TGFBR2, HLA-A, ASAP2, PSD4, HLA-C, SMAD2, HLA-B, HLA-F, SMAP2, CXCR4, SH3KBP1, DNAJC6, EHD1 |
