# Supplementary material for: Deciphering the scalene association among type‐2 diabetes mellitus, prostate cancer, and chronic myeloid leukemia via enrichment analysis of disease‐gene network
Source: Cancer Med. 2019 Apr 1;8(5):2268–77. doi: 10.1002/cam4.1845 (PMC6536925; doi:10.1002/cam4.1845)
Supplement: Supplementary file 4 [file CAM4-8-2268-s004.docx]

**Table S4 The result of enrichment analysis for PCa-related genes**

|  | **Category** | **Term** | **P-Value** | **Genes List** |
| --- | --- | --- | --- | --- |
| 1 | GOTERM_BP_DIRECT | GO:0030198~extracellular matrix organization | 6.60E-10 | F11R, CDH1, DCN, SOX9, COL5A1, VCAM1, SMOC2, LAMB3, FGG, FBLN1, LAMA4, COL14A1, LAMA3, ITGA6, FGA, HPSE2, CD44, SERPINB5, ITGB8, COL6A3, COL1A2, COL6A1, LAMC2, CYR61 |
| 2 | GOTERM_BP_DIRECT | GO:0045944~positive regulation of transcription from RNA polymerase II promoter | 2.76E-06 | FGFR2, RNASEL, HNF1B, HRAS, THRB, ONECUT1, TP63, FGF10, DCN, SOX9, ZBTB38, EPCAM, PHIP, TCF21, ADIRF, FOS, MEIS2, GATA5, MYOCD, NKX3-1, MYC, NFATC3, NR1H4, AKIRIN2, CYR61, MAFG, BMP4, KLF6, AR, EGR2, CTBP2, MYO6, CCNH, RFX6, TBX5, CEBPG, OTX1, MET, IGF1, CDK7, FOSB, TET2, GRHL1, TOX2, ATF3, ITGA6, SFRP2, EBF2, IRF2, WNT7A |
| 3 | GOTERM_BP_DIRECT | GO:0007155~cell adhesion | 1.21E-05 | CCL2, SEMA5A, ALCAM, VCAM1, LAMB3, CD44, ITGB8, COL6A3, COL6A1, SSX2IP, CYR61, SPON1, F11R, CNTNAP5, EFNB1, CD99, EFS, COL5A1, FARP2, THY1, AMBP, RND3, LAMA4, LAMA3, ITGA6, LSAMP, LAMC2, MFAP4, DST |
| 4 | GOTERM_BP_DIRECT | GO:0045893~positive regulation of transcription, DNA-templated | 9.32E-05 | HNF1B, FGF10, TP63, CDH1, CHEK2, SOX9, SEC14L2, PHIP, FOS, MYOCD, NKX3-1, EGF, MYC, NFATC3, RBPJL, BMP4, ZNF281, KLF6, AR, EGR2, RFX6, TBX5, IGF1, BRCA2, CDK7, NOTCH4, SMARCA1, WNT7A, ZFHX3 |
| 5 | GOTERM_BP_DIRECT | GO:0015697~quaternary ammonium group transport | 1.46E-04 | SLC22A4, SLC22A3, SLC22A2, SLC22A1 |
| 6 | GOTERM_BP_DIRECT | GO:0031581~hemidesmosome assembly | 1.57E-04 | LAMB3, LAMA3, ITGA6, LAMC2, DST |
| 7 | GOTERM_BP_DIRECT | GO:0008284~positive regulation of cell proliferation | 1.76E-04 | FGFR2, CLDN7, KRT6A, HRAS, FGFR3, SOX9, CBX8, PTEN, EPCAM, PHIP, HPSE2, MYOCD, NKX3-1, EGF, FGFBP1, MYC, AKIRIN2, AR, IGF1, ATF3, CCND2, SFRP2, BNC1, SDCBP, HBEGF, LAMC2, CTSH |
| 8 | GOTERM_BP_DIRECT | GO:0030335~positive regulation of cell migration | 2.56E-04 | BMP4, HRAS, ONECUT1, ACP5, IGF1, SEMA5A, ITGA6, SEMA6D, F3, SDCBP, HBEGF, LAMC2, CTSH, MYLK, CYR61 |
| 9 | GOTERM_BP_DIRECT | GO:0048754~branching morphogenesis of an epithelial tube | 2.63E-04 | BMP4, HNF1B, MET, FGF10, NKX3-1, EGF |
| 10 | GOTERM_BP_DIRECT | GO:0043066~negative regulation of apoptotic process | 2.64E-04 | CLDN7, UNG, TP63, PPT1, SOX9, PTEN, CD74, EPCAM, PHIP, CD44, ALB, LTF, MYC, NR1H4, DPEP1, CYR61, BMP4, EGR3, IGF1, AMIGO2, CCND2, UCP2, MDM4, PRNP, CTSH, WNT7A |
| 11 | GOTERM_BP_DIRECT | GO:0060349~bone morphogenesis | 4.69E-04 | FGFR2, DHRS3, FGFR3, SFRP2, LTF, ACP5 |
| 12 | GOTERM_BP_DIRECT | GO:0050680~negative regulation of epithelial cell proliferation | 4.96E-04 | FGFR2, BMP4, AR, SFRP2, WFDC1, NKX3-1, SOX9, PTEN |
| 13 | GOTERM_BP_DIRECT | GO:0022617~extracellular matrix disassembly | 5.75E-04 | LAMB3, LAMA3, CD44, KLK2, CAPG, MMP7, CDH1, LAMC2, DCN |
| 14 | GOTERM_BP_DIRECT | GO:0010332~response to gamma radiation | 0.001403 | CCL2, TRIM13, BRCA2, TP63, CHEK2, MYC |
| 15 | GOTERM_BP_DIRECT | GO:1901687~glutathione derivative biosynthetic process | 0.001908 | GSTM1, GSTM2, GSTA2, GSTA4, GSTM5 |
| 16 | GOTERM_BP_DIRECT | GO:0007160~cell-matrix adhesion | 0.002015 | VCAM1, FGG, LYPD3, ITGA6, FGA, SNED1, CD44, ITGB8, FERMT2 |
| 17 | GOTERM_BP_DIRECT | GO:0006749~glutathione metabolic process | 0.002547 | GSTM1, GSTM2, GSTA2, GSTA4, GGT2, GSTM5, DPEP1 |
| 18 | GOTERM_BP_DIRECT | GO:0006366~transcription from RNA polymerase II promoter | 0.002548 | MAFG, EGR2, ONECUT1, CCNH, RFX6, CEBPG, OTX1, TP63, FOSB, CDK7, GRHL1, SOX9, SALL3, TCF21, FOS, MEIS2, ATF3, MYOCD, GATA5, EBF2, IRF2, ZFHX3, MYC, NFATC3, NFX1 |
| 19 | GOTERM_BP_DIRECT | GO:0030199~collagen fibril organization | 0.002624 | FMOD, COL14A1, SFRP2, COL1A2, SERPINH1, COL5A1 |
| 20 | GOTERM_BP_DIRECT | GO:0070371~ERK1 and ERK2 cascade | 0.002665 | IGF1, FGF10, EGF, SOX9, CTSH |
| 21 | GOTERM_BP_DIRECT | GO:0010951~negative regulation of endopeptidase activity | 0.003175 | AMBP, SERPINB6, SERPINB5, SPINT2, SERPINA6, SERPINA5, COL6A3, WFDC1, SERPINH1, SERPINI2 |
| 22 | GOTERM_BP_DIRECT | GO:0072378~blood coagulation, fibrin clot formation | 0.003556 | FBLN1, FGG, FGA |
| 23 | GOTERM_BP_DIRECT | GO:0060442~branching involved in prostate gland morphogenesis | 0.003556 | FGFR2, BMP4, NKX3-1 |
| 24 | GOTERM_BP_DIRECT | GO:0043568~positive regulation of insulin-like growth factor receptor signaling pathway | 0.003591 | PHIP, AR, IGF1, IGFBP5 |
| 25 | GOTERM_BP_DIRECT | GO:0050679~positive regulation of epithelial cell proliferation | 0.004613 | FGFR2, BMP4, HRAS, IGF1, FGF10, SOX9, MYC |
| 26 | GOTERM_BP_DIRECT | GO:0048015~phosphatidylinositol-mediated signaling | 0.005128 | FGFR2, FGFR3, IGF1, HBEGF, FGF10, PI4KB, TNRC6B, EGF, PTEN |
| 27 | GOTERM_BP_DIRECT | GO:0015695~organic cation transport | 0.005506 | SLC22A4, SLC22A3, SLC22A2, SLC22A1 |
| 28 | GOTERM_BP_DIRECT | GO:0090303~positive regulation of wound healing | 0.005506 | HRAS, HBEGF, REG3G, MYLK |
| 29 | GOTERM_BP_DIRECT | GO:0070374~positive regulation of ERK1 and ERK2 cascade | 0.005784 | FGFR2, BMP4, FGG, HRAS, FGFR3, CCL2, FGA, CD44, FGF10, PTEN, FSHR, CD74 |
| 30 | GOTERM_BP_DIRECT | GO:0007219~Notch signaling pathway | 0.007854 | HNF1B, KRT19, ONECUT1, NOTCH4, TP63, SOX9, MYC, NR1H4, SEL1L |
| 31 | GOTERM_BP_DIRECT | GO:0051150~regulation of smooth muscle cell differentiation | 0.008601 | FGFR2, BMP4, MYOCD |
| 32 | GOTERM_BP_DIRECT | GO:0060664~epithelial cell proliferation involved in salivary gland morphogenesis | 0.008601 | FGFR2, FGF10, NKX3-1 |
| 33 | GOTERM_BP_DIRECT | GO:0000122~negative regulation of transcription from RNA polymerase II promoter | 0.008851 | FGFR2, HNF1B, THRB, TP63, CBX2, CBX8, SOX9, TCF21, ZGPAT, MEIS2, HEXIM2, MYOCD, BCL6, SKIL, MYC, AKIRIN2, NR1H4, NFX1, BMP4, ZNF281, CTBP2, FOSB, PKIA, PPP1R13L, ASCL2, ATF3, TDG, IRF2, MDM4, ZFHX3 |
| 34 | GOTERM_BP_DIRECT | GO:0048557~embryonic digestive tract morphogenesis | 0.009347 | FGFR2, TCF21, HNF1B, FGF10 |
| 35 | GOTERM_BP_DIRECT | GO:0007050~cell cycle arrest | 0.009757 | HRAS, STK11, GAS1, SKIL, CDK7, RRAGD, PRNP, DST, MYC, ZFHX3 |
| 36 | GOTERM_BP_DIRECT | GO:0035690~cellular response to drug | 0.010031 | CCL2, NKX3-1, CHEK2, PRNP, MYC, DPEP1, PPP1R14A |
| 37 | GOTERM_BP_DIRECT | GO:0043491~protein kinase B signaling | 0.010608 | CCL2, IGF1, NKX3-1, SOX9, PTEN |
| 38 | GOTERM_BP_DIRECT | GO:0031668~cellular response to extracellular stimulus | 0.010899 | FOS, SLC1A2, ITGA6, SFRP2 |
| 39 | GOTERM_BP_DIRECT | GO:0048286~lung alveolus development | 0.011706 | FGFR2, BMP4, TCF21, MYOCD, IGFBP5 |
| 40 | GOTERM_BP_DIRECT | GO:0032808~lacrimal gland development | 0.011846 | FGFR2, FGF10, SOX9 |
| 41 | GOTERM_BP_DIRECT | GO:0060449~bud elongation involved in lung branching | 0.011846 | FGFR2, BMP4, FGF10 |
| 42 | GOTERM_BP_DIRECT | GO:0015872~dopamine transport | 0.011846 | SLC22A3, SLC22A2, SLC22A1 |
| 43 | GOTERM_BP_DIRECT | GO:0030916~otic vesicle formation | 0.011846 | FGFR2, FGF10, SOX9 |
| 44 | GOTERM_BP_DIRECT | GO:0032355~response to estradiol | 0.012086 | BMP4, FGA, F3, BRCA2, FGF10, WFDC1, PRDM2, WNT7A, PTEN |
| 45 | GOTERM_BP_DIRECT | GO:0007565~female pregnancy | 0.012152 | AMBP, FOS, SLC38A3, UCP2, PSG5, IGF1, FOSB, IGFBP5 |
| 46 | GOTERM_BP_DIRECT | GO:0050790~regulation of catalytic activity | 0.013295 | AR, FXYD3, PPP4R1, BAG2, CBX8, AUP1 |
| 47 | GOTERM_BP_DIRECT | GO:2001237~negative regulation of extrinsic apoptotic signaling pathway | 0.014114 | PHIP, BMP4, AR, ITGA6, IGF1 |
| 48 | GOTERM_BP_DIRECT | GO:0001657~ureteric bud development | 0.014114 | FGFR2, EPCAM, BMP4, TCF21, SDC1 |
| 49 | GOTERM_BP_DIRECT | GO:0001558~regulation of cell growth | 0.014392 | TMEM97, AGTR1, IGFBPL1, STK11, WFDC1, CYR61, IGFBP5 |
| 50 | GOTERM_BP_DIRECT | GO:0010518~positive regulation of phospholipase activity | 0.015538 | FGFR2, FGFR3, CYR61 |
| 51 | GOTERM_BP_DIRECT | GO:0006936~muscle contraction | 0.015573 | FXYD1, ACTG2, ACTA2, COL4A3BP, MYH11, LMOD1, MYOF, MYLK |
| 52 | GOTERM_BP_DIRECT | GO:0007596~blood coagulation | 0.017181 | GGCX, MAFG, PRKAR2B, P2RX4, FGG, FGA, GATA5, F3, SERPINA5, COL1A2, IRF2 |
| 53 | GOTERM_BP_DIRECT | GO:0010838~positive regulation of keratinocyte proliferation | 0.019653 | TP63, FGF10, REG3G |
| 54 | GOTERM_BP_DIRECT | GO:0045669~positive regulation of osteoblast differentiation | 0.019805 | BMP4, SFRP2, LTF, TP63, IGF1, CYR61 |
| 55 | GOTERM_BP_DIRECT | GO:0030574~collagen catabolic process | 0.021060 | COL6A3, COL1A2, MMP7, COL6A1, PHYKPL, COL5A1 |
| 56 | GOTERM_BP_DIRECT | GO:0030155~regulation of cell adhesion | 0.021435 | LAMA4, LAMA3, PPP2CA, SOX9, SERPINI2 |
| 57 | GOTERM_BP_DIRECT | GO:0007517~muscle organ development | 0.023169 | EGR3, SGCG, COL6A3, SGCD, IGF1, HBEGF, ZFHX3 |
| 58 | GOTERM_BP_DIRECT | GO:0042327~positive regulation of phosphorylation | 0.023195 | AR, ITGA6, SDCBP, EGF |
| 59 | GOTERM_BP_DIRECT | GO:0060174~limb bud formation | 0.0241683 | FGFR2, FGF10, SOX9 |
| 60 | GOTERM_BP_DIRECT | GO:0006952~defense response | 0.025135 | KCNN4, CRISP3, LILRA3, TFF3, MX1, CD74 |
| 61 | GOTERM_BP_DIRECT | GO:0042060~wound healing | 0.025518 | BMP4, KLF6, KRT6A, SDC1, TFF3, FGF10, DCN |
| 62 | GOTERM_BP_DIRECT | GO:0002053~positive regulation of mesenchymal cell proliferation | 0.025750 | FGFR2, TP63, SOX9, MYC |
| 63 | GOTERM_BP_DIRECT | GO:0031069~hair follicle morphogenesis | 0.025750 | FGFR2, TP63, FGF10, IGFBP5 |
| 64 | GOTERM_BP_DIRECT | GO:0009887~organ morphogenesis | 0.026750 | FGFR2, HRAS, CCL2, GATA5, THRB, DCN, SYK |
| 65 | GOTERM_BP_DIRECT | GO:0044267~cellular protein metabolic process | 0.027559 | APCS, FGA, KLK3, LTF, IGF1, CTSH, ABCA3, IGFBP5 |
| 66 | GOTERM_BP_DIRECT | GO:0007179~transforming growth factor beta receptor signaling pathway | 0.028021 | F11R, FOS, CCL2, NLK, FERMT2, COL1A2, SKIL |
| 67 | GOTERM_BP_DIRECT | GO:0031018~endocrine pancreas development | 0.028449 | HNF1B, ONECUT1, RFX6, SOX9 |
| 68 | GOTERM_BP_DIRECT | GO:0034116~positive regulation of heterotypic cell-cell adhesion | 0.029061 | FGG, FGA, CD44 |
| 69 | GOTERM_BP_DIRECT | GO:2000773~negative regulation of cellular senescence | 0.029061 | TP63, BCL6, TERT |
| 70 | GOTERM_BP_DIRECT | GO:0045165~cell fate commitment | 0.030714 | FGFR2, GATA5, ONECUT1, GAS1, WNT7A |
| 71 | GOTERM_BP_DIRECT | GO:0007568~aging | 0.031202 | VCAM1, FOS, LONP1, CCL2, UCP2, F3, MMP7, IGF1, DCN, PTEN, IGFBP5 |
| 72 | GOTERM_BP_DIRECT | GO:2000352~negative regulation of endothelial cell apoptotic process | 0.031293 | SEMA5A, FGG, FGA, TERT |
| 73 | GOTERM_BP_DIRECT | GO:0021983~pituitary gland development | 0.031293 | BMP4, FGF10, CDH1, SRD5A1 |
| 74 | GOTERM_BP_DIRECT | GO:0042476~odontogenesis | 0.031293 | FGFR2, BMP4, SDC1, COL1A2 |
| 75 | GOTERM_BP_DIRECT | GO:0043388~positive regulation of DNA binding | 0.031293 | MYOCD, CEBPG, IGF1, EGF |
| 76 | GOTERM_BP_DIRECT | GO:0060333~interferon-gamma-mediated signaling pathway | 0.031308 | VCAM1, CD44, TRIM8, IRF2, HLA-DPB1, GBP1 |
| 77 | GOTERM_BP_DIRECT | GO:0046777~protein autophosphorylation | 0.031538 | FGFR2, FGFR3, STK11, NLK, LMTK2, CDK12, WNK2, CHEK2, SYK, THY1 |
| 1 | KEGG_PATHWAY | hsa04512:ECM-receptor interaction | 6.88E-05 | LAMA4, SDC1, LAMB3, LAMA3, ITGA6, CD44, ITGB8, COL6A3, COL1A2, COL6A1, LAMC2, COL5A1 |
| 2 | KEGG_PATHWAY | hsa04151:PI3K-Akt signaling pathway | 1.53E-04 | FGFR2, HRAS, FGFR3, STK11, MET, IGF1, LPAR3, FGF10, PTEN, COL5A1, LAMB3, YWHAG, LAMA4, LAMA3, ITGA6, CCND2, ITGB8, PPP2CA, COL6A3, COL1A2, COL6A1, LAMC2, EGF, MYC, SYK |
| 3 | KEGG_PATHWAY | hsa05200:Pathways in cancer | 1.82E-04 | FGFR2, HRAS, FGFR3, GNA11, FGF10, LPAR3, CDH1, PTEN, FOS, AGTR1, LAMB3, NKX3-1, HHIP, EGF, MYC, BMP4, AR, CTBP2, KLK3, MET, BRCA2, IGF1, LAMA4, LAMA3, ITGA6, LAMC2, WNT7A |
| 4 | KEGG_PATHWAY | hsa04510:Focal adhesion | 2.03E-04 | HRAS, MET, IGF1, FLNC, PTEN, COL5A1, LAMB3, LAMA4, LAMA3, ITGA6, ITGB8, CCND2, COL6A3, COL1A2, COL6A1, LAMC2, EGF, MYLK |
| 5 | KEGG_PATHWAY | hsa04550:Signaling pathways regulating pluripotency of stem cells | 0.001271 | BMP4, FGFR2, HRAS, FGFR3, ONECUT1, OTX1, IGF1, POU5F1B, MAPK13, SKIL, WNT7A, MYC, ZFHX3 |
| 6 | KEGG_PATHWAY | hsa04974:Protein digestion and absorption | 0.001515 | KCNN4, COL14A1, KCNK5, COL6A3, COL1A2, COL6A1, CELA2B, CELA2A, COL5A1, CTRL |
| 7 | KEGG_PATHWAY | hsa05219:Bladder cancer | 0.008220 | HRAS, FGFR3, HBEGF, CDH1, EGF, MYC |
| 8 | KEGG_PATHWAY | hsa04514:Cell adhesion molecules (CAMs) | 0.012458 | ALCAM, VCAM1, F11R, CLDN7, SDC1, ITGA6, ITGB8, CD99, CDH1, CLDN11, HLA-DPB1 |
| 9 | KEGG_PATHWAY | hsa05146:Amoebiasis | 0.016674 | GNAL, LAMA4, LAMB3, LAMA3, SERPINB6, GNA11, COL1A2, LAMC2, COL5A1 |
| 10 | KEGG_PATHWAY | hsa04115:p53 signaling pathway | 0.017068 | CCND2, SERPINB5, CD82, IGF1, CHEK2, MDM4, PTEN |
| 11 | KEGG_PATHWAY | hsa05215:Prostate cancer | 0.018878 | FGFR2, AR, HRAS, KLK3, IGF1, NKX3-1, EGF, PTEN |
| 12 | KEGG_PATHWAY | hsa05218:Melanoma | 0.022138 | HRAS, MET, IGF1, FGF10, CDH1, EGF, PTEN |
| 13 | KEGG_PATHWAY | hsa04972:Pancreatic secretion | 0.024777 | CEL, PNLIPRP1, PNLIPRP2, CELA2B, CELA2A, SLC4A4, CTRL, SCTR |
| 14 | KEGG_PATHWAY | hsa04530:Tight junction | 0.025989 | EPB41L2, F11R, CLDN7, HRAS, SHROOM2, PPP2CA, MYH11, CLDN11, TJP3, PTEN |
| 15 | KEGG_PATHWAY | hsa04310:Wnt signaling pathway | 0.027083 | SFRP5, CTBP2, CCND2, SFRP2, NLK, PRICKLE2, MMP7, WNT7A, NFATC3, MYC |
| 16 | KEGG_PATHWAY | hsa05205:Proteoglycans in cancer | 0.045848 | HRAS, SDC1, HPSE2, CD44, MAPK13, MET, IGF1, HBEGF, DCN, FLNC, WNT7A, MYC |
| 17 | KEGG_PATHWAY | hsa05230:Central carbon metabolism in cancer | 0.047422 | FGFR2, HRAS, FGFR3, MET, PTEN, MYC |
| 18 | KEGG_PATHWAY | hsa05222:Small cell lung cancer | 0.047557 | LAMA4, LAMB3, LAMA3, ITGA6, LAMC2, PTEN, MYC |
