# Supplementary material for: Deciphering the scalene association among type‐2 diabetes mellitus, prostate cancer, and chronic myeloid leukemia via enrichment analysis of disease‐gene network
Source: Cancer Med. 2019 Apr 1;8(5):2268–77. doi: 10.1002/cam4.1845 (PMC6536925; doi:10.1002/cam4.1845)
Supplement: Supplementary file 5 [file CAM4-8-2268-s005.docx]

**Table S5 The comparison of enrichment analysis for disease-related genes**

| **Diseases** | **Functions** | **Pathways** |
| --- | --- | --- |
| T2DM vs CML vs PCa | Negative regulation of apoptotic process;  Negative regulation of transcription from RNA polymerase II promoter RNA; | **----** |
| T2DM vs CML | Response to drug;  Fat cell differentiation;  Response to glucose;  Cytoskeleton organization; | FoxO signaling pathway |
| T2DM vs PCa | Endocrine pancreas development; | ---- |
| CML vs PCa | Negative regulation of endothelial cell apoptotic process;  Positive regulation of cell migration;  Positive regulation of cell proliferation;  Interferon-gamma-mediated signaling pathway;  Positive regulation of cell death;  Aging;  Positive regulation of DNA binding;  Response to estradiol;  Cell adhesion;  Negative regulation of extrinsic apoptotic signaling pathway via death domain receptors;  Single organismal cell-cell adhesion;  Regulation of cell growth;  Positive regulation of transcription from RNA polymerase II promote; Extracellular matrix disassembly;  Extracellular matrix organization;  Wound healing;  Positive regulation of transcription,  DNA-templated;  Cell cycle arrest;  Negative regulation of endopeptidase activity;  Collagen fibril organization;  Negative regulation of cell migration;  Negative regulation of cell proliferation;  Positive regulation of osteoblast differentiation;  Collagen catabolic process;  Blood coagulation;  Cellular response to extracellular stimulus;  Positive regulation of ERK1 and ERK2 cascade. | Pathways in cancer;  Focal adhesion;  Cell adhesion molecules (CAMs);  Proteoglycans in cancer;  ECM-receptor interaction;  Melanoma;  PI3K-Akt signaling pathway;  Amoebiasis;  Bladder cancer; |
