# Supplementary material for: Deciphering the scalene association among type‐2 diabetes mellitus, prostate cancer, and chronic myeloid leukemia via enrichment analysis of disease‐gene network
Source: Cancer Med. 2019 Apr 1;8(5):2268–77. doi: 10.1002/cam4.1845 (PMC6536925; doi:10.1002/cam4.1845)
Supplement: Supplementary file 6 [file CAM4-8-2268-s006.docx]

**Table S6 The top 10 modules in T2DM-related gene network**

| **Cluster** | **Score (Density*#Nodes)** | **Nodes** | **Edges** | **Node IDs** |
| --- | --- | --- | --- | --- |
| 1 | 16.062 | 33 | 257 | myt1, irs1, neurod1, nkx6-3, tcf7l2, pax4, prox1, cdkn2a, hhex, onecut2, pparg, hsf4, slc30a8, dusp9, st18, cdkal1, adcy5, kcnq1, srr, dach1, wfs1, shox2, dusp8, insm1, pax6, hmga2, rfx6, tspan8, pdx1, chchd9, lmx1a, tnpo1, mlxipl |
| 2 | 15 | 15 | 105 | akt3, btg1, c6orf62, c7orf60, kifap3, fbxo11, arid4a, cops2, tmem59, snord46, gltscr2, rbms1, rpl29, gstm2, c15orf32 |
| 3 | 13 | 13 | 78 | fabp6, tnfaip6, plg, fxyd4, gcgr, slc12a1, slc6a3, ndufa4l2, kng1, nphs2, enpp3, slc13a3, nptx2 |
| 4 | 10.1 | 21 | 101 | st3gal5, rngtt, alkbh1, aco1, c10orf57, sephs1, hcls1, plau, plaur, ifitm3, ttc39b, klhdc6, pdap1, sgcd, mapk14, st3, alkbh3, lyn, nudt14, cnn2, eepd1 |
| 5 | 10 | 10 | 45 | st8sia4, vav1, apc, man2a1, trim17, ccng1, ap3b1, lmnb1, or5ac2, or1j2 |
| 6 | 9 | 9 | 36 | btn1a1, olah, irf7, irf5, arg2, cel, folr1, stat3, nf-kappab |
| 7 | 9 | 9 | 36 | klk7, pdgfd, ctnna2, fbxo39, npy2r, kcnk17, pcdhb2, znf132, dcdc2 |
| 8 | 9 | 9 | 36 | ube4b, cbfa2t3, gabrb3, cntnap4, col18a1, nipa1, ptpn6, aldh2, erbb2 |
| 9 | 9 | 9 | 36 | trak1, tcf12, lmo7, ank3, acsl5, osbpl1a, scin, ugp2, chek1 |
| 10 | 8.533 | 16 | 64 | cxxc5, znf783, fzd1, cyb5r2, htr1a, syn2, nuak1, axpc1, rpl39l, phactr3, slc6a4, aplp2, syne2, bnc1, lmx1b, bdnf |
