# Supplementary material for: Deciphering the scalene association among type‐2 diabetes mellitus, prostate cancer, and chronic myeloid leukemia via enrichment analysis of disease‐gene network
Source: Cancer Med. 2019 Apr 1;8(5):2268–77. doi: 10.1002/cam4.1845 (PMC6536925; doi:10.1002/cam4.1845)
Supplement: Supplementary file 7 [file CAM4-8-2268-s007.docx]

**Table S7 The top 10 modules in CML-related gene network**

| **Cluster** | **Score** | **Nodes** | **Edges** | **Node IDs** |
| --- | --- | --- | --- | --- |
| 1 | 33.522 | 68 | 1123 | gbx2, klhl35, tmem30b, fam78a, fam62c, efemp2, dpys, chn2, dhrs3, chn1, derl3, arhgap1, ddit4l, cpt1c, col2a1, akt1, cnnm1, clic5, barhl1, abhd9, pik3r2, prdx2, racgap1, arhgap29, gmip, krt31, stard13, arhgap26, arhgap25, arhgap24, hoxa9, arhgap23, arhgap22, arhgap21, arhgap20, cdkn2a, arhgap19, arhgap18, arhgap17, unc5c, arhgap15, cdc42, srgap2, tmem22, srgap3, srgap1, slc27a3, arhgap12, rec8, arhgap10, rax, arhgap9, rasd1, arhgap8, ptprg, dlc1, pax6, arhgap6, hoxd13, arhgap5, hoxd12, pik3r1, hoxd11, arhgap4, tlx2, gprasp1, prlhr, tgfb2 |
| 2 | 24 | 24 | 276 | spp1, utp11l, tnfsf10, nr3c1, bnip1, tnfrsf1b, col4a3, cd5, bbc3, casp9, tiaf1, tp73, birc8, tnf, casp7, tp53, stk17a, znf443, cdkn1a, cebpg, birc3, sst, tnfrsf25, hmi |
| 3 | 21 | 21 | 210 | cox5b, cox5a, taldo1, uqcrh, pgk1, sdha, gpi, ndufs1, ndufb10, fbp1, eno1, aldoa, ogdh, ndufa5, idh3a, idh2, dlst, atp5h, atp5b, atp5a1, cox2 |
| 4 | 20.718 | 40 | 404 | ptpn13, prox1, angpt2, tnfsf13b, ddx3x, cd40lg, creb3l3, cd28, stmn1, mcam, spink1, atp6v0c, ceacam1, cxcr4, ccr6, pak1, il23r, kiaa0101, ccr7, il26, arntl, rorc, il22, cldn10, cap2, mmp2, gli2, runx1, ccl20, il17a, hk2, furin, cxcr6, rhoa, atf5, ctsh, ltb, faslg, map3k4, id1 |
| 5 | 15 | 15 | 105 | gltscr2, snord46, tmem59, cops2, arid4a, kifap3, c7orf60, c6orf62, btg1, akt3, c15orf32, gstm2, rpl29, fbxo11, rbms1 |
| 6 | 13.462 | 94 | 626 | faah, sox21, tspyl5, ubxn10, foxi2, gstm5, dlx5, slc13a5, col4a2, il4, znf217, bcat1, jun, fabp7, b3gnt5, myo5b, egr1, grasp, ervk2, msx2, hspb1, efna5, ocln, hspa4l, adcy5, fos, erc2, cnrip1, cdo1, magi1, ptk7, rspo1, spry1, gipc2, npy, pcdhb3, foxf1, vegfa, irf4, me1, pcdhb17, pcdha1, pcdh9, pfn2, daam1, prickle4, mn1, celsr3, bmp1, znf382, dsh, sall1, gna14, mmp10, zscan18, fgf5, ikzf1, vim, hccs, gadd45a, tmeff2, cacna1g, spg20, fsd1, bcl2a1, bcr, snca, elovl4, selplg, cebpb, sfrp1, dpysl4, ccl2, mal, znf177, ina, gfpt2, fbn1, irak3, dclk1, tnfrsf10c, wisp1, oas1, egf, neurog1, hcc, sdc2, lbx1, il8, st8sia1, c3orf14, c7orf13, bst2, slc6a15 |
| 7 | 10.805 | 88 | 470 | znf157, cnn2, dazl, nf-kappab, nudt14, axpc1, alkbh3, alkbh1, rac1, mapk14, btk, pdap1, lxn, syk, ptn, lyn, crp, gp6, ros1, nox1, abcb1, plg, gcgr, abcg2, cdh1, olfm4, slc13a3, nphs2, abcg1, kng1, abcc4, slc12a1, abcg4, lilra2, fxyd4, stat3, fabp6, abcd2, tgfb1i1, enpp3, abcc10, ndufa4l2, abcc9, abcc6, tnfaip6, nptx2, slc6a3, ikbke, stat5b, cdk6, hmga2, abcb6, il1r1, ptk2, ccr2, wnt16, ngf, s100a10, cd68, s100a6, il18, pik3ca, adamts4, has1, dnajc6, vps26b, htatip2, inhba, abca8, rabgef1, ifitm3, mitf, col1a2, tmem127, st3gal5, st3, htr2c, sts, pkc, il6, sephs1, pou3f4, mmd2, itsn1, atg16l1, myc, aco1, msn |
| 8 | 8.111 | 145 | 584 | xpo6, bmi1, golm1, ern1, cfhr3, prdm16, pdgfa, cdh9, fgf2, met, kdr, igf1, tead4, brd2, flt1, igf1r, chek1, erbb2, aph1b, ncstn, tbp, psenen, mgs, psen2, igfbp6, lamc2, il27, bace2, ncam1, alpp, bace1, psen1, silv, app, mcm4, cd3e, itgal, srm, hmox1, s100b, ctgf, tgfb1, s100a8, s100a13, igkv2d-18, s100a1, timm8a, gpx1, faf1, atf3, gstp1, fat, plaur, ttc39b, c10orf57, rngtt, ndrg1, eepd1, abcc2, sgcd, klhdc6, hcls1, egfr, loc400927, postn, loc203547, ttll1, ttpal, mgat3, kif5c, serhl, slc25a17, api5, poldip3, dtl, stat5a, itgb2, aldh3a2, foxp2, rps6ka2, mdfic, crim1, csmd3, snx9, scin, acsl5, gstm1, lmo7, txnrd1, col14a1, eif4g2, ugp2, cat, il2ra, ank3, mirn21, wnt1, snord87, trak1, ptgdr, itgb7, sod1, osbpl1a, prima1, itgad, tcf12, tbx3, pcdh8, foxp3, thbs2, fas, cdh4, socs3, cyr61, sfrp2, usp29, lrrk2, cds1, ralyl, raly, l3mbtl3, prtn3, ptpn23, lats2, bag5, jub, bcl2, bmp2, strbp, lyve1, ssh2, dock8, il8ra, hoxc6, sgsm3, plau, adam10, epas1, e2f8, mycbp2, cdca5, top2a, myo15a, sstr1, yap1 |
| 9 | 8 | 8 | 28 | dhrs4, akr1c4, akr1c1, akr1c2, akr1b10, cbr3, akr1b1, cbr1 |
| 10 | 8 | 8 | 28 | sfrs10, atp1b1, slc30a9, kctd9, ckap5, 3.6.1.8, psme2, paics |
