# Supplementary material for: Deciphering the scalene association among type‐2 diabetes mellitus, prostate cancer, and chronic myeloid leukemia via enrichment analysis of disease‐gene network
Source: Cancer Med. 2019 Apr 1;8(5):2268–77. doi: 10.1002/cam4.1845 (PMC6536925; doi:10.1002/cam4.1845)
Supplement: Supplementary file 8 [file CAM4-8-2268-s008.docx]

**Table S8 The top 10 modules in CML-related gene network**

| **Cluster** | **Score** | **Nodes** | **Edges** | **Node IDs** |
| --- | --- | --- | --- | --- |
| 1 | 30.885 | 62 | 942 | hoxa9, pax6, hoxd13, cdkn2a, prlhr, gbx2, klhl35, fam78a, fam62c, lgr4, efemp2, sspn, dpys, adcy3, il27, dhrs3, pomc, atxn2l, derl3, sema3g, sh2b1, ddit4l, itih4, spns1, cpt1c, elp4, cln3, col2a1, immp1l, sult1a2, cnnm1, agrn, sult1a1, prdx2, clic5, six5, barhl1, map2k5, abhd9, bdnf, rps10, unc5c, psip1, tmem22, pmaip1, tlx2, hoxc12, tufm, tgfb2, scgb3a1, slc27a3, mgat1, rec8, nr1h3, ptpmt1, rax, rasd1, ptprg, hoxd12, hoxd11, tmem30b, gprasp1 |
| 2 | 21.703 | 149 | 1606 | ccr6, clu, rara, cr1, bin1, pitx2, il22, dlx4, lhx1, b4galnt2, egfr, gas7, cdkn1a, bnc1, cd5, rab11a, casp9, casp7, brca1, bnip1, prima1, bcr, shd, runx3, mest, dab2ip, c4b, rbp1, hmi, nr3c1, spp1, sst, tp73, mme, stk17a, hoxa11, mef2c, tiaf1, tnfrsf25, fzd9, sall1, tnfrsf1b, fgf2, tnfsf10, fabp3, utp11l, znf443, col4a3, cebpg, klk10, rab27a, il17a, sytl5, bcl11b, birc8, rab17, ing1, rab10, spock1, rab9a, hand2, rab8a, adcy5, rab7a, furin, rspo1, birc3, rab5a, cxcr6, sox14, rab4a, faslg, dlx1, rab2a, arntl, sox11, rab1a, atf5, rab6a, runx1, ctsh, sox9, rab3a, ltb, map3k4, mtg1, ptpn13, tp53, app, cd40lg, rab37, cd28, rab28, mcam, rab25, ceacam1, gstm2, rab23, il23r, rab20, il26, esr1, ltbp2, rab18, rorc, serpina5, trip4, rfx1, tnf, cass4, lhx2, fermt2, apc, zcwpw1, sgk1, mll, txndc3, tnfsf13b, pax3, inpp5d, en1, ptk2b, tff1, hla-drb1, six3, hla-drb5, sorl1, rassf1, cd2ap, nfe2l3, abca7, msi1, ms4a6a, hdac9, magea1, ms4a4a, epha1, ptgs2, cd33, picalm, tfap2a, cd44, bbc3, nr2f2, akt1, thy1, clathrin, ccl20, gstp1 |
| 3 | 20 | 20 | 190 | saps2, pdia2, phyh, osbpl8, thbs4, sh3bgr, enosf1, gosr1, stat2, gpd1, sma4, dci, recql, orc2l, pde8a, per2, camta1, trappc6a, rasgrp1, c21orf33 |
| 4 | 19 | 19 | 171 | zbtb4, sfrs2, rpl41, cct2, cabc1, mthfd2, rps15a, naca, rps12, fzd7, rpl7, dbh, bst1, rpl6, nckap1, slc25a19, itgb1, rps6, flna |
| 5 | 18 | 18 | 153 | apoa4, apoa1, nr1h2, ch25h, cyp46a1, lrp8, vldlr, lrp1, abca2, lpa, apod, apoc3, ldlr, apoc2, abca1, soat1, apoc1, apoe |
| 6 | 16.062 | 33 | 257 | mlxipl, cyp19a1, insm1, cyp11a1, hsf4, cyp51a1, rfx6, hmgcr, hmgcs1, lmx1a, star, gas6, rbp4, inhba, aldh1b1, neurod1, amh, cyp17a1, insl3, cdh1, cpa1, gata4, pdx1, shox2, dach1, st18, hsd17b7, prox1, pax4, onecut2, nkx6-3, tnpo1, myt1 |
| 7 | 12.207 | 59 | 354 | slc12a1, fxyd4, col14a1, fabp6, col12a1, enpp3, col11a1, ndufa4l2, col6a3, tnfaip6, col3a1, nptx2, slc6a3, chek1, serpine1, pthlh, ephb1, spink5, mapk14, serpinb11, krt3, clca4, tgm3, crnn, il8, mal, s100a7a, col1a1, nell2, mmp1, cxcl10, bcl2, bax, il10, timp1, tnfsf13, cxcl16, cxcl1, il12b, il1a, atr, ddr1, mx2, ifit3, il6, ifit1, hspb1, isg15, plg, il1b, gcgr, nos2, tnfa, lcn2, slc13a3, ctgf, nphs2, cdc2, kng1 |
| 8 | 12 | 12 | 66 | cdh13, efemp1, dapk1, dlec1, mlh1, lsamp, mgmt, cmtm3, sfrp1, fhit, rassf5, opcml |
| 9 | 12 | 12 | 66 | wdr60, rph3al, spsb2, mfap2, fam163a, il17ra, nt5dc2, a2ml1, ep400, cst9l, oca2, kcnq2 |
| 10 | 11 | 11 | 55 | ndufs6, paip1, sdha, tars, basp1, nnt, osmr, pols, skp2, rad1, brd9 |
