# Supplementary material for: Deciphering the scalene association among type‐2 diabetes mellitus, prostate cancer, and chronic myeloid leukemia via enrichment analysis of disease‐gene network
Source: Cancer Med. 2019 Apr 1;8(5):2268–77. doi: 10.1002/cam4.1845 (PMC6536925; doi:10.1002/cam4.1845)
Supplement: Supplementary file 9 [file CAM4-8-2268-s009.docx]

**Table S9 The GO enrichment analysis of overlapping modules in T2DM, CML and PCa (P<0.05)**

| **Diseases** | **Term** | **Genes** | **P-value** |
| --- | --- | --- | --- |
| T2DM  vs  CML | GO:0006508~proteolysis | TMEM59, ST14, DPP8, DPP9, DPP4 | 0.0022 |
|  | GO:0006366~transcription from RNA polymerase II promoter | MAF, MAFG, COPS2, ARID4A, MNT | 0.0029 |
|  | GO:0010388~cullin deneddylation | COPS2, COPS8 | 0.0107 |
|  | GO:0008285~negative regulation of cell proliferation | BTG1, KIFAP3, MNT, COPS8 | 0.0120 |
|  | GO:0000715~nucleotide-excision repair, DNA damage recognition | COPS2, COPS8 | 0.0270 |
|  | GO:0022008~neurogenesis | COPS2, LLGL2 | 0.0500 |
| CML  vs  PCa | GO:0034765~regulation of ion transmembrane transport | KCNMA1, CACNA1H, SCN4A | 0.0037 |
|  | GO:0050872~white fat cell differentiation | CTBP1, CTBP2 | 0.0108 |
|  | GO:0019079~viral genome replication | CTBP1, CTBP2 | 0.0116 |
|  | GO:0086010~membrane depolarization during action potential | CACNA1H, SCN4A | 0.0231 |
